# Analysis and Presentation of Cumulative Antimicrobial Susceptibility Test Data – The Influence of Different Parameters in a Routine Clinical Microbiology Laboratory

**DOI:** 10.1371/journal.pone.0147965

**Published:** 2016-01-27

**Authors:** Rebekka Kohlmann, Sören G. Gatermann

**Affiliations:** 1 Department of Medical Microbiology, Ruhr-University Bochum, Bochum, Germany; 2 Institute of Medical Laboratory Diagnostics (IML) Bochum GmbH, Bochum, Germany; California Department of Public Health, UNITED STATES

## Abstract

**Introduction:**

Many clinical microbiology laboratories report on cumulative antimicrobial susceptibility testing (cAST) data on a regular basis. Criteria for generation of cAST reports, however, are often obscure and inconsistent. Whereas the CLSI has published a guideline for analysis and presentation of cAST data, national guidelines directed at clinical microbiology laboratories are not available in Europe. Thus, we sought to describe the influence of different parameters in the process of cAST data analysis in the setting of a German routine clinical microbiology laboratory during 2 consecutive years.

**Material and Methods:**

We developed various program scripts to assess the consequences ensuing from different algorithms for calculation of cumulative antibiograms from the data collected in our clinical microbiology laboratory in 2013 and 2014.

**Results:**

One of the most pronounced effects was caused by exclusion of screening cultures for multi-drug resistant organisms which decreased the MRSA rate in some cases to one third. Dependent on the handling of duplicate isolates, i.e. isolates of the same species recovered from successive cultures on the same patient during the time period analyzed, we recorded differences in resistance rates of up to 5 percentage points for *S*. *aureus*, *E*. *coli* and *K*. *pneumoniae* and up to 10 percentage points for *P*. *aeruginosa*. Stratification by site of care and specimen type, testing of antimicrobials selectively on resistant isolates, change of interpretation rules and analysis at genus level instead of species level resulted in further changes of calculated antimicrobial resistance rates.

**Conclusion:**

The choice of parameters for cAST data analysis may have a substantial influence on calculated antimicrobial resistance rates. Consequently, comparability of cAST reports from different clinical microbiology laboratories may be limited. We suggest that laboratories communicate the strategy used for cAST data analysis as long as national guidelines for standardized cAST data analysis and reporting do not exist in Europe.

## Introduction

In the era of increasing antimicrobial resistance worldwide but reduced emphasis on antibiotic development by pharmaceutical manufacturers, the need for antimicrobial resistance surveillance and the importance of a prudent use of antibiotics are now widely recognized. Since empiric antimicrobial therapy is guided by several considerations including local resistance pattern of commonly isolated bacteria, many clinical microbiology laboratories regularly provide cumulative antimicrobial susceptibility test (cAST) data. Reliable cumulative antibiograms may help to avoid both choice of ineffective antibiotics and excessive prescription of broad-spectrum antibiotics in case individual culture results are not (yet) available. Furthermore, cAST reports may be used to compare antimicrobial resistance rates across institutions, to monitor resistance trends over time within an institution, and to plan and evaluate antibiotic stewardship interventions. However, the usefulness of cumulative antibiograms largely depends on standardized antimicrobial susceptibility testing as well as transparent and consistent generation of cAST reports across different clinical microbiology laboratories.

Although the Clinical and Laboratory Standards Institute (CLSI) has developed a guideline regarding analysis and presentation of cAST data [1, 2], summarized in Table 1, considerable variation in cAST data analysis was still present in the USA almost 10 years after first publication of this guideline with only 47% of the responding laboratories having adopted all of the standards recommended by the CLSI [3].

**Table 1:** Main CLSI recommendations for analysis and presentation of cAST data.

Generate cumulative antibiograms at least annually.
Consider only species with antimicrobial susceptibility testing data for at least 30 isolates to guarantee statistical validity of the estimates.
Calculate cumulative antibiograms preferably at species level. In addition, for *Staphylococcus aureus*, calculate separate cumulative antibiograms for all isolates and for the subset of methicillin-resistant isolates.
Calculate the percentage susceptible per species/antibiotic combination, and do not include isolates with intermediate susceptibility.
Include only diagnostic isolates, but not isolates from surveillance and screening cultures or from non-patient sources.
Include only the first isolate of a given species per patient per analysis period. Comment: The methods for handling of duplicate isolates (i.e., isolates of the same species recovered from successive cultures on the same patient during the time period analyzed) are diverse: (1) All isolates strategy: all isolates of a given species collected during the time period analyzed are considered equally. (2) First isolate strategy: only the first isolate of a given species per patient per analysis period is considered irrespective of body site, specimen type, antimicrobial susceptibility profile, or other phenotypic characteristics (= CLSI proposal). (3) Episode-based strategy: duplicate isolates are included when the minimal interval of time between their recovery was n (e.g., 5, 10, or 30) days (i.e., the first isolate of a given species per patient is included, then further isolates are excluded for n days until the next isolate is included again); unfortunately, there is no agreed consensus on the definition of an episode, and multiple different time intervals have been proposed. (4) Antibiogram-based strategy: duplicate isolates are selected with respect to their antimicrobial susceptibility profile, with a variety of possible inclusion criteria, for example, considering every isolate with a deviating antimicrobial susceptibility profile per patient (with regard to either key antimicrobials or to the whole antimicrobial susceptibility testing panel), selecting the most resistant or the most susceptible isolate per patient, or calculating a weighted average of a patient’s susceptibility test results for each species/antibiotic combination. (5) Individual variations / combinations of the above listed strategies: for example, method (2) or (3) but including only isolates from normally sterile body sites; combination of (3) and (4), i.e., considering the first isolate of successive episodes only in case of a different antimicrobial susceptibility profile.
Report results only for antibiotics that are routinely tested. If your laboratory applies selective reporting rules whereby specific antimicrobial agents are tested on merely a subset of isolates, do not report those supplemental results. Comment: Selective testing policies are common, including: (1) body site-specific testing (e.g., nitrofurantoin tested only for urinary tract isolates), (2) second-line / cascade testing (i.e., second-line antimicrobials, such as tigecycline and colistin, tested merely on species with resistance to first-line antibiotics), (3) prescribing-specific testing (i.e., only those antimicrobials tested which are requested or currently used for treatment).
Generate separate reports for each health care facility served by your laboratory, ideally with additional data stratification according to the current clinical needs of the facility. Comment: A variety of subanalyses may be conducted provided that sufficient numbers of non-duplicate isolates have been tested to allow statistical validity of resistance estimates for the subgroups, including stratification by: (1) time-point of culturing (community-acquired vs. health care-associated infection), (2) patient location (specific hospital wards, such as intensive care units, or less specific designations, such as inpatients vs. outpatients), (3) patient population (e.g., pediatric patients, cancer patients), (4) specimen type or body site from which the specimen was obtained (e.g., blood and other normally sterile samples, urine, respiratory tract specimens, skin swabs), (5) organism’s resistance characteristics (e.g., separate reports for methicillin-resistant vs. methicillin-susceptible *Staphylococcus aureus*).
Acknowledge changes in antimicrobial susceptibility testing procedure by an explanatory note.

In Europe, the guidelines of the European Committee on Antimicrobial Susceptibility Testing (EUCAST) ensure standardization of antimicrobial susceptibility testing [4], but a recommendation for cAST data analysis in the local routine clinical microbiology laboratory is lacking, apart from one document focusing global surveillance rather than local routine diagnostics [5]. Thus, inconsistencies in the methods used to generate cAST reports may be even more pronounced in Europe, and comparability of cAST reports to those of previous years or of other institutions may be limited.

Unfortunately, many clinicians are completely unaware of how the choice of parameters for cAST data analysis can shift antimicrobial resistance rates on the cumulative antibiogram reported by the local routine clinical microbiology laboratory. Therefore, the present study was performed to assess the consequences ensuing from various algorithms for calculation of cumulative antibiograms in a German routine clinical microbiology laboratory. Whereas previous studies merely addressed the influence of few selected parameters (e.g., [6–12]), we intended a comprehensive evaluation of multiple influencing parameters on one single dataset across two observation periods.

## Material and Methods

### Setting

Our clinical microbiology laboratory serves 16 hospitals representing both urban and rural settings from the Bochum area in Germany with more than 4,600 beds overall. The hospitals are mainly tertiary-care teaching hospitals including most facilities of the University Hospital of Ruhr-University Bochum.

### Routine data collection and analysis

Antimicrobial susceptibility testing is performed according to the EUCAST recommendations [4], using Vitek^®^2 (bioMérieux), disk diffusion, and E-tests. Appropriate antimicrobial agents for susceptibility testing are selected after taking into consideration the organism, hospital formulary, and site of infection, respectively. Antimicrobial susceptibility testing results are stored in a locally developed database together with species identification (mainly achieved by MALDI-TOF Mass Spectrometry), quantification, additional tests (e.g., the double-disk synergy test for extended spectrum β-lactamase (ESBL) detection), and clinical data. The latter includes limited patient information (name, date of birth and sex, but no detailed clinical histories), specimen collection date, and sample source (hospital and ward, body site, specimen type), as provided by the treating clinicians when submitting samples for routine microbiology analyses. Antimicrobial susceptibility testing data are always validated by clinical microbiologists prior to reporting.

Our laboratory annually provides cAST reports, listing the total number of tested isolates per species, and for each species/antibiotic combination the number and percentage of susceptible and resistant isolates. For our routine cAST, we exclude isolates derived from surveillance and screening cultures, and we consider duplicate isolates according to an episode-based strategy with an interval of 10 days (as defined in Table 1). Usually, we calculate overall cumulative antibiograms for all species with at least 30 isolates, and hospital-specific cumulative antibiograms for the 15 most common species, respectively. Further analyses, e.g. ward-specific analyses, are performed on request.

### Study-specific analyses

Antimicrobial susceptibility testing results collected in two consecutive years (2013 and 2014) were analyzed using various program scripts to assess the consequences ensuing from different algorithms for calculation of cumulative antibiograms, as detailed below in the respective results and discussion sections. For study-specific analyses of the data obtained during routine patient care, respective antimicrobial susceptibility testing results were selected from the laboratory database, and calculations were performed using locally developed software, with identifying patient information being removed prior to publication. The study was approved by the local ethics committee. Results are shown for selected species and selected antibiotics. In the graphs and tables, antibiotics are abbreviated as follows: penicillin = PEN, oxacillin = OXA, ampicillin = AMP, ampicillin/sulbactam = SAM, piperacillin = PIP, piperacillin/tazobactam = TZP, cefuroxime = CXM, cefotaxime = CTX, ceftazidime = CAZ, cefepime = FEP, imipenem = IPM, meropenem = MEM, gentamicin = GEN, tobramycin = TOB, sulfamethoxazole/trimethoprim = SXT, erythromycin = ERY, clindamycin = CLI, tetracycline = TET, tigecycline = TGC, ciprofloxacin = CIP, levofloxacin = LVX, moxifloxacin = MXF, fosfomycin = FOF, vancomycin = VAN, and rifampicin = RIF.

## Results and Discussion

### Handling of screening cultures

Because screening cultures are primarily collected for the purpose of determining if a patient is harboring particular multi-resistant pathogens and not as part of the clinical evaluation of a patient’s illness, methods that selectively isolate resistant organisms are typically used. Thus, the inclusion of isolates recovered from screening cultures may heavily weight estimates toward higher rates of resistance.

**Fig 1:**
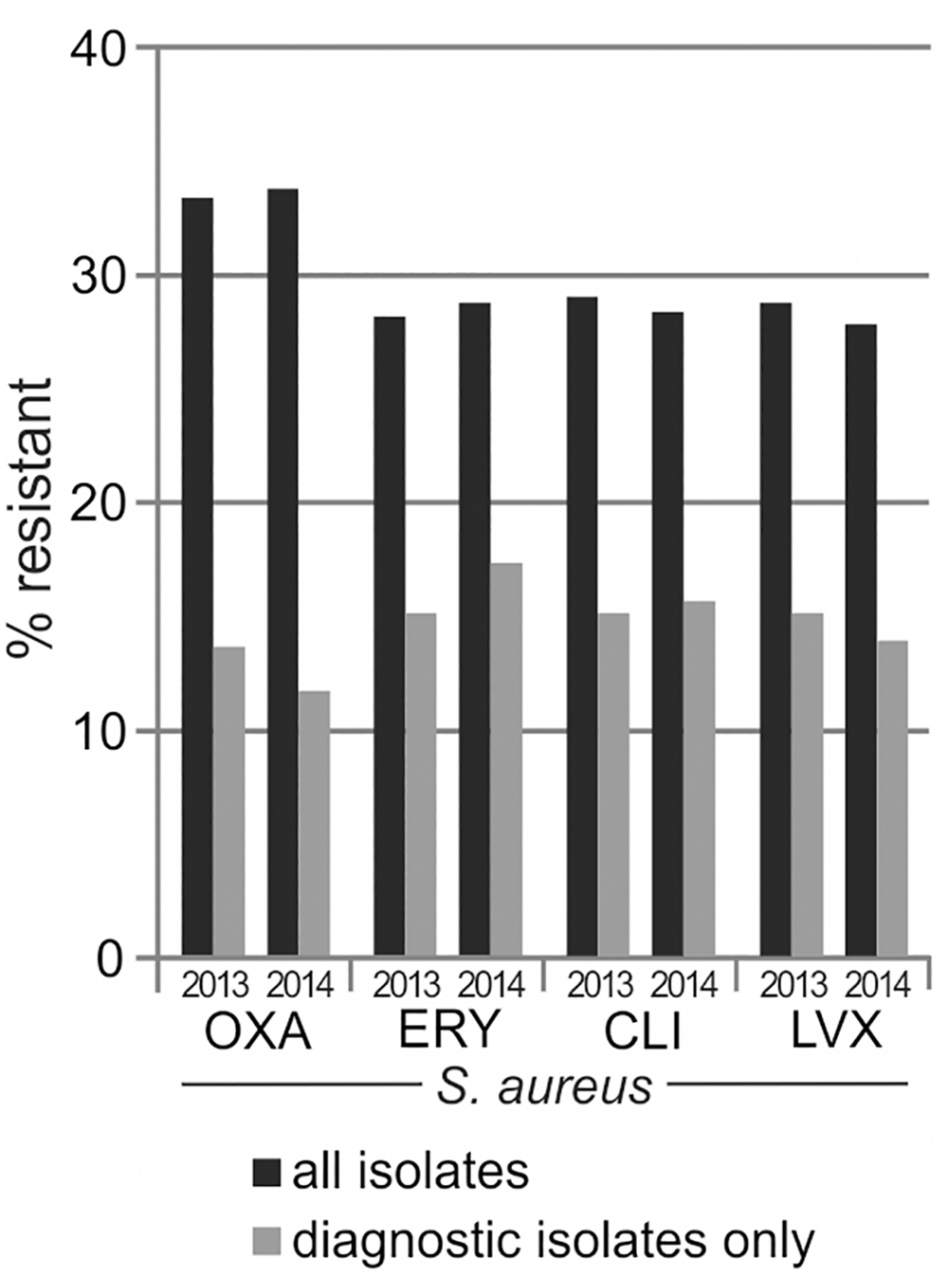
Resistance estimates dependent on the handling of screening isolates. Resistance rates were calculated either with inclusion (black columns) or exclusion (grey columns) of screening isolates, as described in the text. Further details are given in the supporting information (S1 Table).

For analyzing the effect of screening isolate handling, we included *Staphylococcus aureus* antimicrobial susceptibility testing results from one selected hospital which aimed at 100% admission screening rate for methicillin-resistant *Staphylococcus aureus* (MRSA). MRSA screening was performed using selective culture media with subsequent antimicrobial susceptibility testing of all *Staphylococcus aureus* isolates grown on these media. Using our standard procedure for duplicate isolate removal, we calculated cumulative antibiograms for *Staphylococcus aureus* with either inclusion or exclusion of screening isolates. Screening isolates accounted for 25% of total *Staphylococcus aureus* isolates, and their inclusion increased MRSA percentage by 20 to 22 percentage points (Fig 1). Considerable increases of resistance rates were also observed for macrolides, lincosamides and fluoroquinolones because of common cross-resistance in MRSA isolates. For data on further selected species/antibiotic combinations and the number of included isolates, please refer to the supporting information (S1 Table).

Our data are in line with previous studies showing an increase in the MRSA percentage with the inclusion of screening cultures; however, the magnitude of the effect varied dependent on the study setting with increases by 2 to 15 percentage points [11, 12]. Variations across different studies are not surprising because the effect of screening isolate removal depends on the extent and method of screening. As our data are based on a hospital aiming at 100% MRSA admission screening (high percentage of screening samples) and on the use of selective media (over-representation of resistant isolates), the inclusion of screening isolates led to a strong increase in resistance rates, and we would expect less pronounced increases in other settings. However, we would recommend exclusion of screening cultures, as we did for all further analyses shown in this study.

Of note, whereas screening was mainly performed for MRSA in the past, increasing attention is now paid to multi-resistant Gram-negative bacteria. Thus, an inclusion of screening cultures will probably significantly affect resistance rates not only of *Staphylococcus aureus* but also of Gram-negative rods in the future.

### Handling of duplicate isolates

The inclusion of duplicate isolates (as defined in Table 1) may bias resistance estimates in favor of the findings in those patients who are cultured most frequently. Multiple methods for exclusion of duplicate isolates have been proposed (Table 1). However, we believe that antibiogram-based strategies are less feasible for a routine clinical microbiology laboratory because they require sophisticated computer programming, are prone to errors in case of biological variability in antimicrobial susceptibility test results, and uncertainties as how to rate duplicate isolates with only minor discordances (e.g., I→R or S→I transitions) may occur. Thus, we restricted our analyses to the all isolates, first isolate and episode-based approaches.

**Fig 2:**
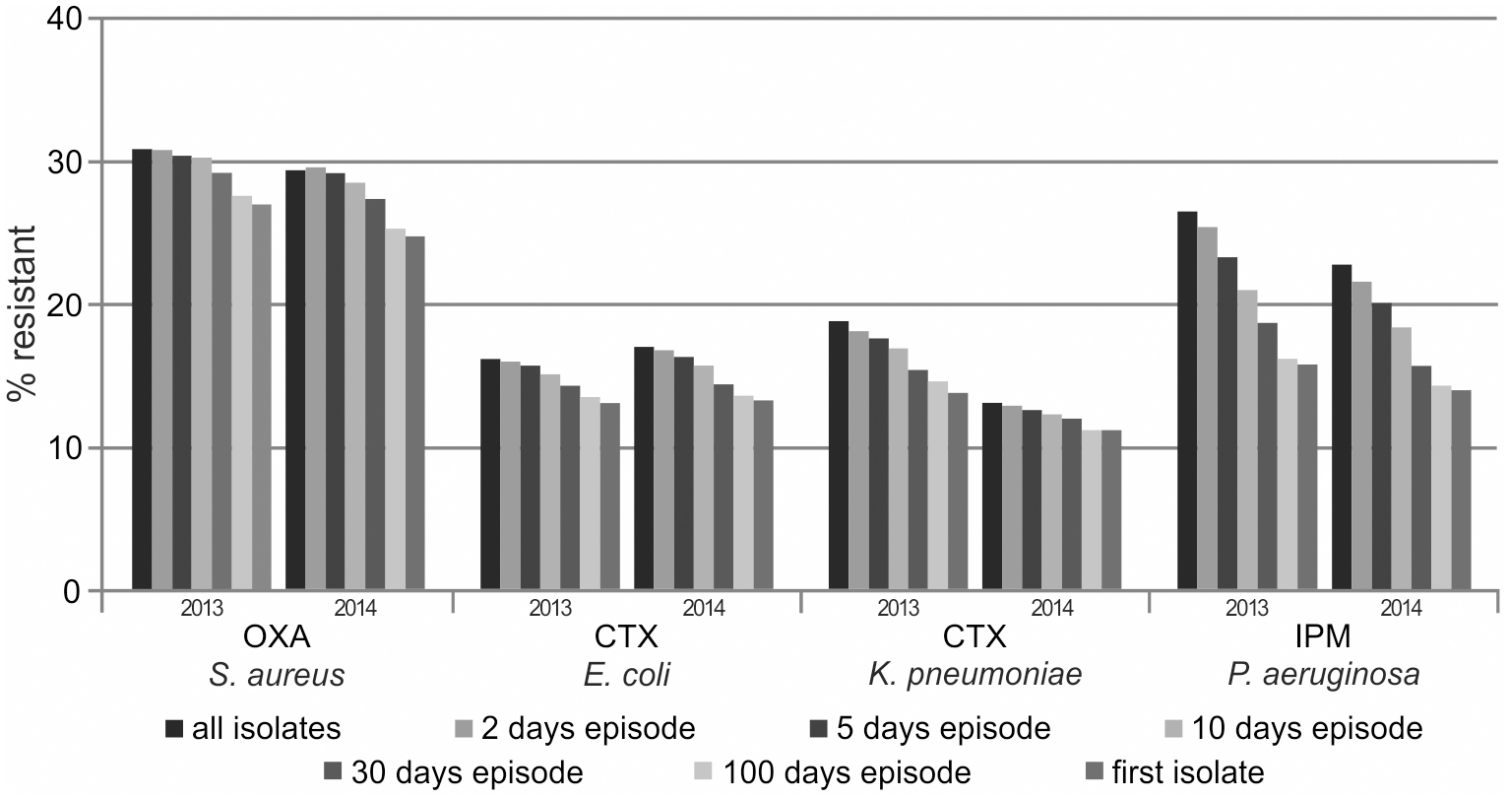
Resistance estimates dependent on the method of duplicate isolate removal. Resistance rates were calculated using different methods of duplicate isolate removal, as described in the text. Further details are given in the supporting information (S2 Table).

For analyzing the effect of duplicate isolate removal, we considered all diagnostic isolates recovered in our laboratory (i.e., screening isolates were excluded), and generated cAST reports based on the all isolates strategy, different episode-based strategies (minimal interval of time: 2, 5, 10, 30 or 100 days) and the first isolate strategy. In Fig 2, results are shown exemplarily for oxacillin-resistance in *Staphylococcus aureus*, cefotaxime-resistance in *Escherichia coli* and *Klebsiella pneumoniae*, and imipenem-resistance in *Pseudomonas aeruginosa*. Please refer to the supporting information for the number of included isolates and data on further selected species/antibiotic combinations (S2 Table). Of note, as with the exclusion of screening isolates, the effect of duplicate isolate removal was similar in the two analyzed observation periods indicating reliability of our data. A considerable percentage of isolates was removed from calculation, at most 44.9% using the first isolate approach, with duplicate isolates being most common for *Pseudomonas aeruginosa* (S2 Table). For pathogens that can be acquired preclinically and nosocomially, the duplicate isolate removal decreased resistance rates by up to 10 percentage points for *Pseudomonas aeruginosa* and up to 5 percentage points for *Staphylococcus aureus*, *Escherichia coli* and *Klebsiella pneumoniae* (Fig 2, S2 Table). The more the predefined minimal interval for inclusion of duplicate isolates was lengthened, the more isolates were removed and the lower were the calculated resistance rates. Of note, this effect was not observed for bacteria which are usually only pre-clinically acquired such as *Streptococcus agalactiae* (S2 Table).

The phenomenon of increasing resistance rates with the inclusion of duplicate isolates may be explained as follows: Patients with long hospital stays or with underlying chronic diseases requiring frequent antimicrobial treatment, and patients with treatment failure are more likely to be cultured repeatedly. It is biologically plausible that these subgroups of patients have a higher probability of harboring resistant bacteria which may be acquired in hospital or selected during antimicrobial treatment. In addition, patients with complicated bacterial infections (e.g., caused by multidrug-resistant organisms) are often monitored for bacterial clearance. Thus, the inclusion of duplicate isolates may lead to a bias toward higher rates of resistance. In contrast, the first isolate strategy tends to stress the antimicrobial susceptibility test results for bacteria that are present at patient admission; therefore, subsequent emergence of resistance may be missed, resulting in an overly optimistic view of the percentage of resistance. Typically, the effect of duplicate isolate removal is more pronounced for species which are usually hospital-acquired and which are prone to emergence of resistance (e.g., *Pseudomonas aeruginosa*).

Studies on the effect of duplicate isolate removal may lead to varying results, depending not only on the method used for duplicate isolate removal, but also on local endemicity, sampling practice and patient demographics. For example, local policies of frequent repetitive sampling, hospitalization instead of outpatient treatment, severity of infection, underlying diseases and patient age may affect the number of duplicate isolates. Therefore, comparability of different studies is limited and it is not possible to confidently predict the effect of a duplicate isolate removal. However, previous studies mainly showed an decrease in resistance rates with the exclusion of duplicate isolates similar to our findings, albeit analyses were often limited to a single species or used fewer methods for duplicate isolate removal: Regarding the MRSA percentage, decreases by up to 35 percentage points were found in different studies, and the effect depended on duplicate isolate removal strategy, patient population, observation period, study site and sample type [7, 9, 13–15]. Regarding other species/antibiotic combinations, decreases in resistance rates by up to 5 percentage points were found for urinary tract isolates [16], up to 12 and 18 percentage points for *Escherichia coli* and *Pseudomonas aeruginosa* isolates [10] and up to 19 and 11 percentage points for *Klebsiella pneumoniae* and *Acinetobacter baumannii* isolates [15]. Picking one exemplary study for a more detailed comparison [12], Shannon et al. showed a decrease by up to 20 percentage points for methicillin-resistance of *Staphylococcus aureus* and gentamicin-resistance of *Klebsiella* spp., depending on the strategy used for duplicate isolate removal. The more pronounced effect in comparison to our findings may be explained by the percentage of duplicates which was about twice as high as in our study. In contrast, Shannon et al. did not find an effect on resistance rates of outpatient *Escherichia coli* isolates, probably because of the lower rate of resistance and the lower likelihood to undergo repeat culturing among outpatients compared to hospitalized patients. Interestingly, whereas the aforementioned studies and our own findings documented merely decreasing resistance rates with the exclusion of duplicate isolates, others observed inconsistent results with both decreases and increases, depending on the species/antibiotic combination and the duplicate isolate removal strategy [11, 17–20].

Interestingly, in two studies comparing multiple strategies for duplicate isolate removal including antibiogram-based calculations [12, 14], resistance estimates calculated with the first isolate or episode-based strategies were within the range bounded by the most resistant and the most susceptible interpretations per patient and, thus, may be reflective of the true rates of resistance. In contrast, the all isolates strategy resulted in resistance rates higher than those obtained by including only the most resistant isolate per patient which argues against the application of the all isolates method. However, the first isolate strategy may underestimate resistance rates, especially in the inpatient setting, as it led to resistance estimates closely approaching the most susceptible interpretations per patient. Thus, episode-based strategies may be better suited. Accordingly, it was published in 2001 that cAST reports based on a 30 days episode strategy reflect resistance rates among hospital-acquired infections [21]. Because the average length of hospital stay has continuously decreased in Germany from 14.0 days in 1991 to 7.5 days in 2013 [22], we believe that at present a shorter time interval should be used for discrimination of different infectious episodes. Thus, we consider the 10 days episode strategy to be the first choice for the inpatient setting. However, there may be no single "correct" way to eliminate duplicate isolates, and other calculation strategies may be selected in case of other individual reporting needs (e.g., the first isolate strategy for guiding empirical therapy of initial infections in outpatients). It may also be discussed whether species-specific time intervals should be selected in episode-based approaches because the decrease in resistance estimates occurred later for *Staphylococcus aureus* than for *Pseudomonas aeruginosa* (Fig 2, S2 Table). As we are not aware of a biological reason for this finding, we still propose to follow the 10 days episode strategy.

### Data stratification according to acquisition of infection

In addition to an overall cAST report, subanalyses based on additional data stratification may be conducted according to the current clinical needs of the facility (Table 1). We show the effect of some of these stratification options, starting with the comparison of community-acquired and hospital-acquired isolates using the time interval between MRSA admission screening and isolate recovery as the differentiation criterion. A more precise differentiation between community-acquired and hospital-acquired isolates would require detailed clinical data which is beyond the means of a routine clinical microbiology laboratory.

**Fig 3:**
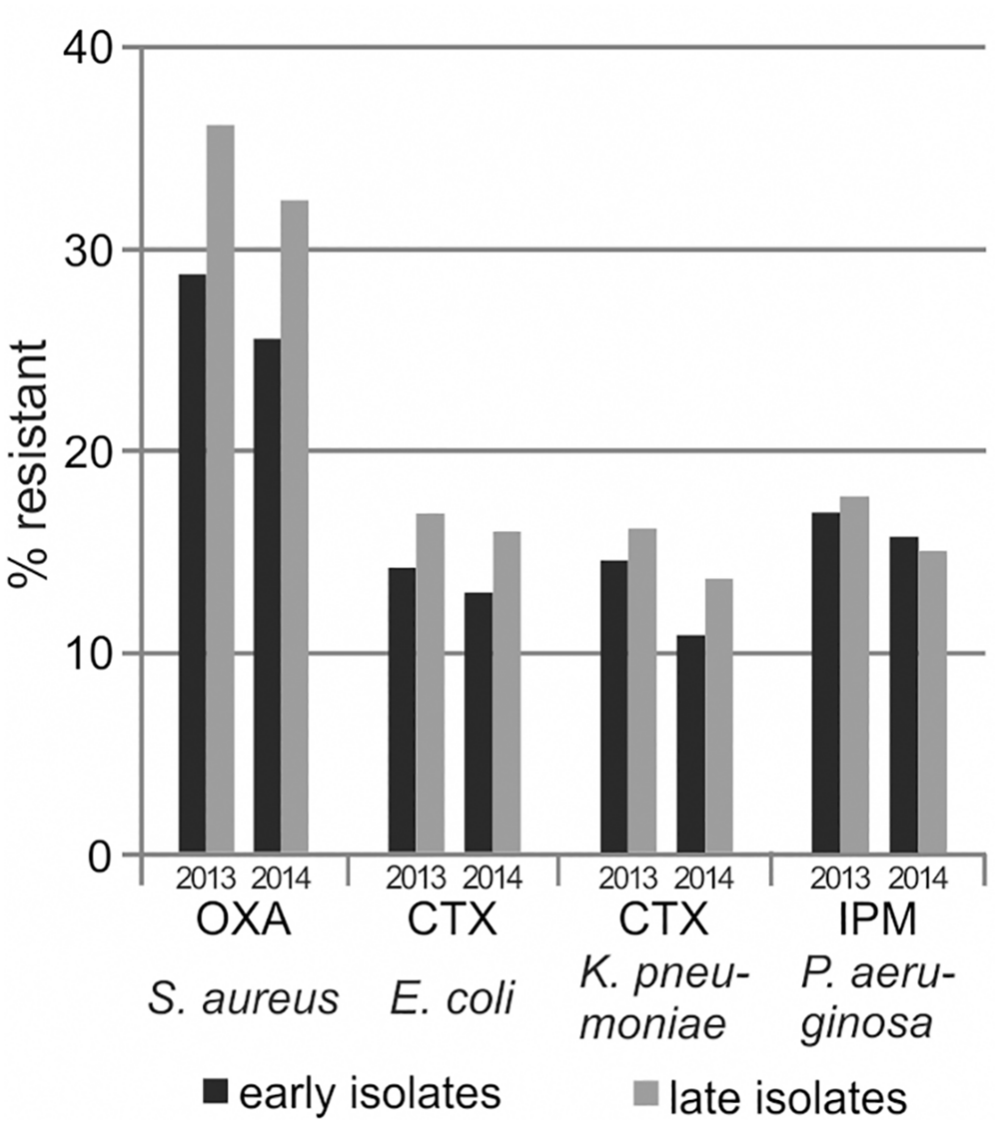
Resistance estimates dependent on the time-point of isolate recovery. Resistance rates were calculated with respect to early isolates (black columns) or late isolates (grey columns), as described in the text. Further details are given in the supporting information (S3 Table).

We considered the antimicrobial susceptibility test results for isolates in case MRSA admission screening was performed for the respective patient. Isolates recovered during the first 3 days after MRSA screening (i.e., sampling during the first 48 h of hospitalization) were considered community-acquired (early isolates), the remaining isolates hospital-acquired (late isolates). For calculation of resistance rates, screening isolates were excluded and only the first early or the first late isolate were considered per patient. Exemplary results are shown in Fig 3; for the number of included isolates and data on further selected species/antibiotic combinations, please refer to the supporting information (S3 Table). As expected, for pathogens that can be acquired both preclinically and nosocomially, late isolates exhibited up to 8 percentage points higher resistance rates than early isolates, whereas a uniform trend was not detected for pathogens that are usually acquired merely preclinically.

Even though one may assume that the resistance rates of early and late isolates could be reflected by the resistance estimates calculated using the first isolate and the all isolates strategy, respectively, results were not comparable in our study. For example, differences in resistance estimates were most pronounced for *Staphylococcus aureus* when comparing early and late isolates, but most pronounced for *Pseudomonas aeruginosa* when comparing the first isolate and the all isolates strategy. In addition, resistance rates of late isolates exceeded resistance estimates based on the all isolates strategy for *Staphylococcus aureus*, but resembled resistance estimates calculated with the 30 days episode strategy for *Pseudomonas aeruginosa*. These observations may be explained by differences between *Staphylococcus aureus* and *Pseudomonas aeruginosa* regarding the main time-points of pathogen acquisition and emergence of resistance during the course of the hospitalization.

To our knowledge, only few previous studies compared resistance rates in community-acquired vs. hospital-acquired isolates, each showing most pronounced differences for *Pseudomonas aeruginosa* with up to 16 percentage points higher resistance rates in hospital-acquired isolates [10, 23, 24]. Smaller differences of up to 9 percentage points were observed for *Escherichia coli* and *Staphylococcus aureus*, and previous antimicrobial treatment and hospitalization within the last 30 days were identified as further risk factors for higher resistance rates.

Similarly, resistance rates were shown to be higher in hospitalized patients and nursing home residents compared to outpatients, with differences of up to 23 percentage points depending on the species/antibiotic combination and the study setting [14, 16, 25, 26]. However, this effect may be age-dependent, as in one study increased resistance rates in inpatient isolates were observed merely in pediatric patients and adults for *Escherichia coli* and merely in adults and elderly people for *Staphylococcus aureus* [27].

### Data stratification according to patient location

It is widely accepted that separate cAST reports should be generated for each health care facility served by a laboratory because local resistance rates may be highly variable (e.g., shown by [28]). However, the aggregation of data across a single hospital may also obscure differences among patient populations and hospital wards. Thus, a hospital-wide cumulative antibiogram may be misleading.

**Fig 4:**
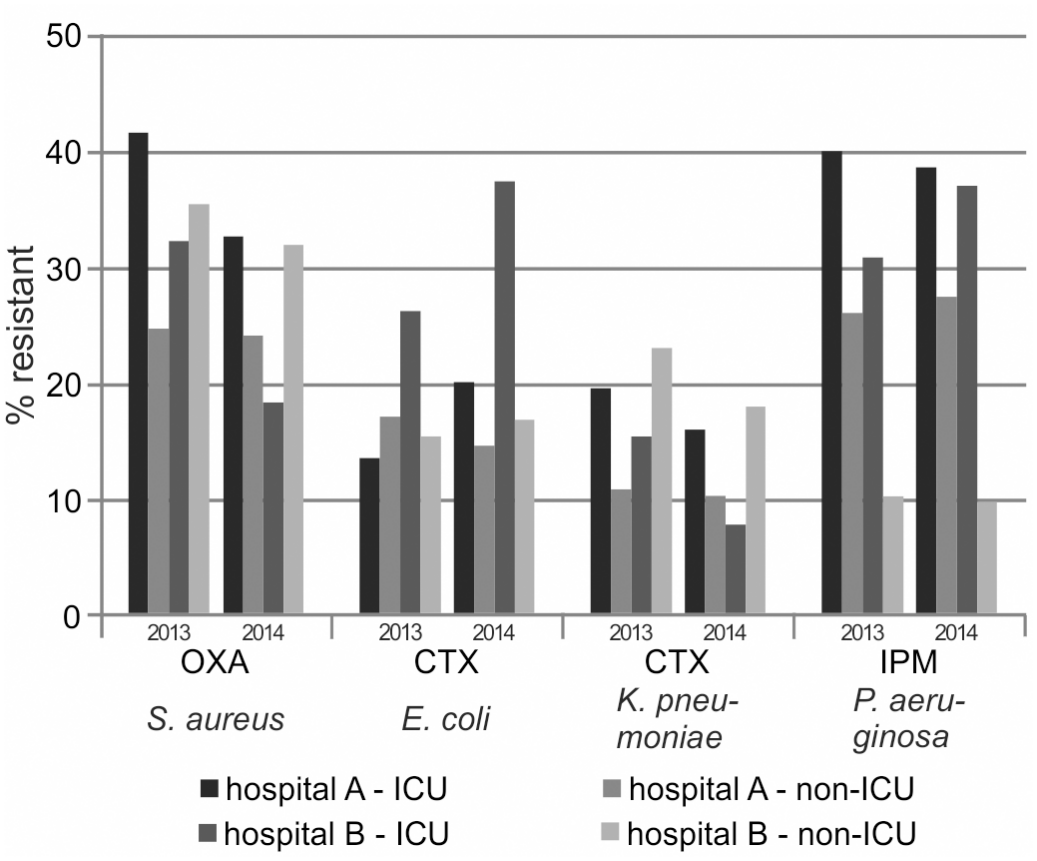
Resistance estimates dependent on the patient location. Resistance rates were calculated with data stratification according to the patient location (hospital and ward), as described in the text. Further details are given in the supporting information (S4 Table).

We evaluated the effect of a stratification according to patient location, both at the inter-hospital level by comparing cAST data of two randomly selected hospitals served by our laboratory (named A and B) and at the intra-hospital level by comparing cAST data of intensive care units (ICUs) and non-ICU-wards for each of these two hospitals. For calculation of resistance rates, all isolates recovered from the two selected hospitals were considered apart from screening isolates, and duplicate isolates were removed according to a 10 days episode strategy. Exemplary results are shown in Fig 4; for the number of included isolates and data on further selected species/antibiotic combinations, please refer to the supporting information (S4 Table). Resistance rates varied broadly and without predictable patterns both across hospitals and within a single hospital. Regarding the inter-hospital comparison, hospital A exhibited partly higher and partly lower resistance rates compared to hospital B with no consistent pattern. With respect to the intra-hospital comparison, it is generally believed that antimicrobial resistance is more prevalent in ICUs than in other areas of the hospital. However, this held unambiguously true only for *Pseudomonas aeruginosa* isolates with up to 27 percentage points higher resistance rates in the ICUs. In contrast, a uniform pattern was not observed for *Escherichia coli*, *Staphylococcus aureus* and *Klebsiella pneumoniae* isolates. Interestingly, when comparing the two observation periods, the trends were similar in most but not in all cases (e.g., cefotaxime-resistance of *Escherichia coli* in the ICU compared to the non-ICU wards of hospital A was lower in 2013 but higher in 2014).

Several previous studies compared unit-specific antibiograms for ICU- and non-ICU-patients. Focusing on merely the MRSA percentage [7, 9, 14] or *Pseudomonas aeruginosa* resistance rates [25, 29], the authors typically observed higher values in the ICUs, namely 2 to 30 percentage points higher depending on the study setting. However, studies showing ICU / non-ICU comparisons for a broader spectrum of species/antibiotic combinations described rather inconsistent differences just as we did [10, 23, 30–32].

Further unit-specific analyses have been provided with respect to pediatric patients and elderly people. For pediatric units, researchers observed mainly lower resistance rates for a number of species/antibiotic combinations in comparison to the hospital-wide antibiogram [23, 24, 27, 29, 33]. Results were most pronounced for fluoroquinolones with 10 to 25 percentage points lower resistance rates in patients ≤18 years. For geriatric units, less consistent results with variable deviations from the hospital-wide antibiogram were found including higher MRSA percentages and fluoroquinolones resistance rates [27, 32]. In addition, the inclusion of highly specialized units with distinct antimicrobial resistance patterns may bias hospital-wide antibiograms, as was shown for cystic fibrosis-derived *Pseudomonas aeruginosa* isolates [6]. Of note, the range of resistance rates may be very high across one single hospital, with up to 46 percentage points for *Pseudomonas aeruginosa* in one study [10].

### Data stratification according to specimen type

Cumulative antibiograms combine not only data from various units but also from various body sites. Whereas isolates from cultures taken from normally sterile sites (e.g., blood cultures) are often the causes of infections, isolates from other body sites may also represent colonization.

**Fig 5:**
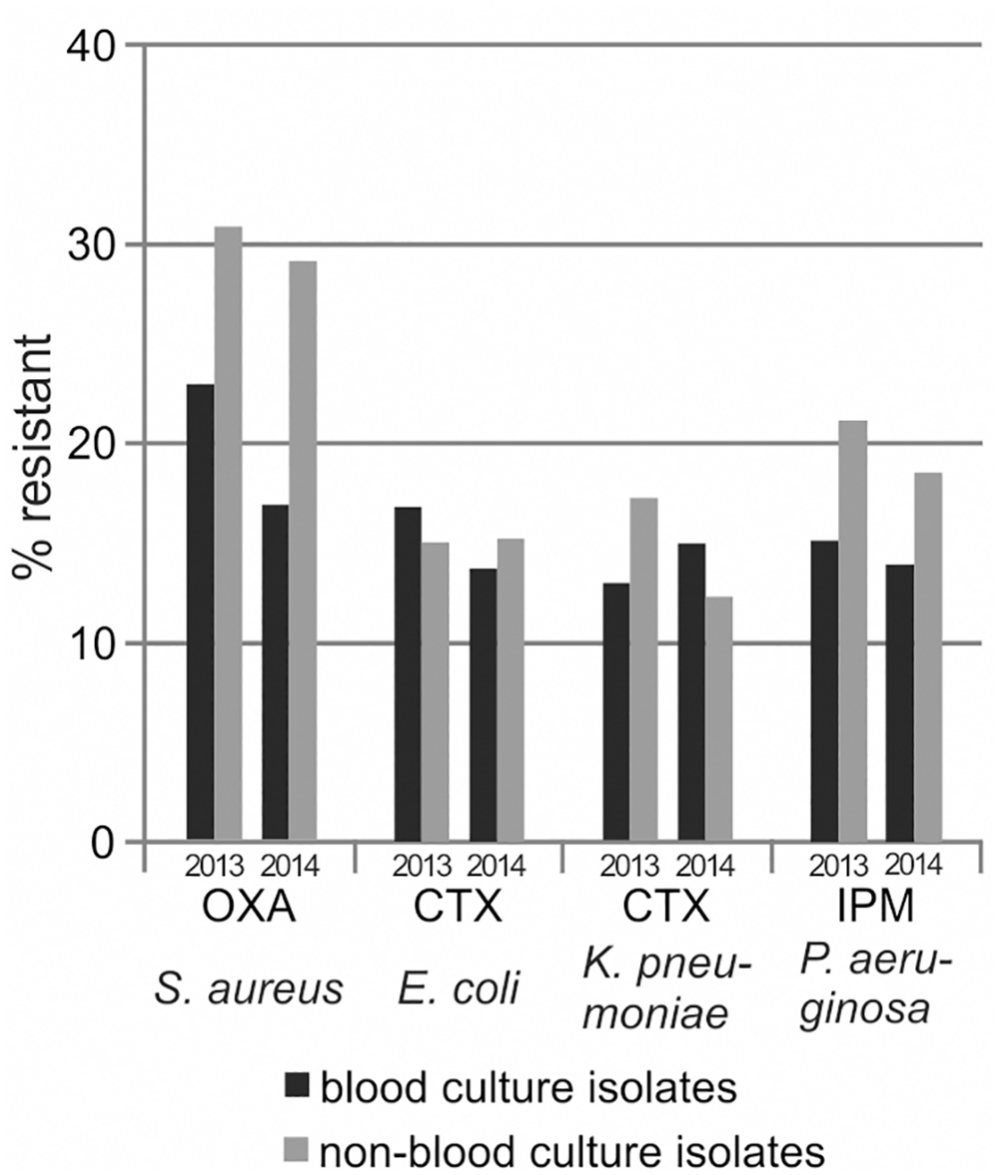
Resistance estimates dependent on the specimen type. Resistance rates were calculated with data stratification according to the specimen type (black columns: isolates recovered from blood cultures, grey columns: isolates recovered from other body sites), as described in the text. Further details are given in the supporting information (S5 Table).

We compared resistance rates of blood culture isolates and other isolates. For calculation of resistance rates, we considered all isolates recovered in our laboratory, after exclusion of screening isolates and with duplicate isolate removal according to a 10 days episode strategy. Exemplary results are shown in Fig 5; for the number of included isolates and data on further selected species/antibiotic combinations, please refer to the supporting information (S5 Table). Whereas resistance rates of *Staphylococcus aureus* and *Pseudomonas aeruginosa* were considerably lower in blood culture isolates across both observation periods (up to 14.5 percentage points), differences were inconsistent and less pronounced for *Escherichia coli* and *Klebsiella pneumoniae*.

In previous studies, resistance rates were often similar for different specimen types or showed merely unpredictable deviations [7, 10, 23, 29]. However, one study found mainly higher resistance rates of blood culture isolates for several examined species/antibiotic combinations [32] which is in contrast to our data, whereas another study also found lower resistance rates in *Staphylococcus aureus* isolates from normally sterile body sites [13].

### Data stratification according to organism’s resistance characteristics

Whereas the CLSI guideline recommends generating a separate report for methicillin-resistant *Staphylococcus aureus*, it otherwise discourages the reporting of resistance rates for antimicrobials tested only on a subset of isolates (Table 1). However, selective testing policies are common in many laboratories. Thus, we exemplify the effect of selective testing policies on the cAST report with respect to second-line testing.

**Fig 6:**
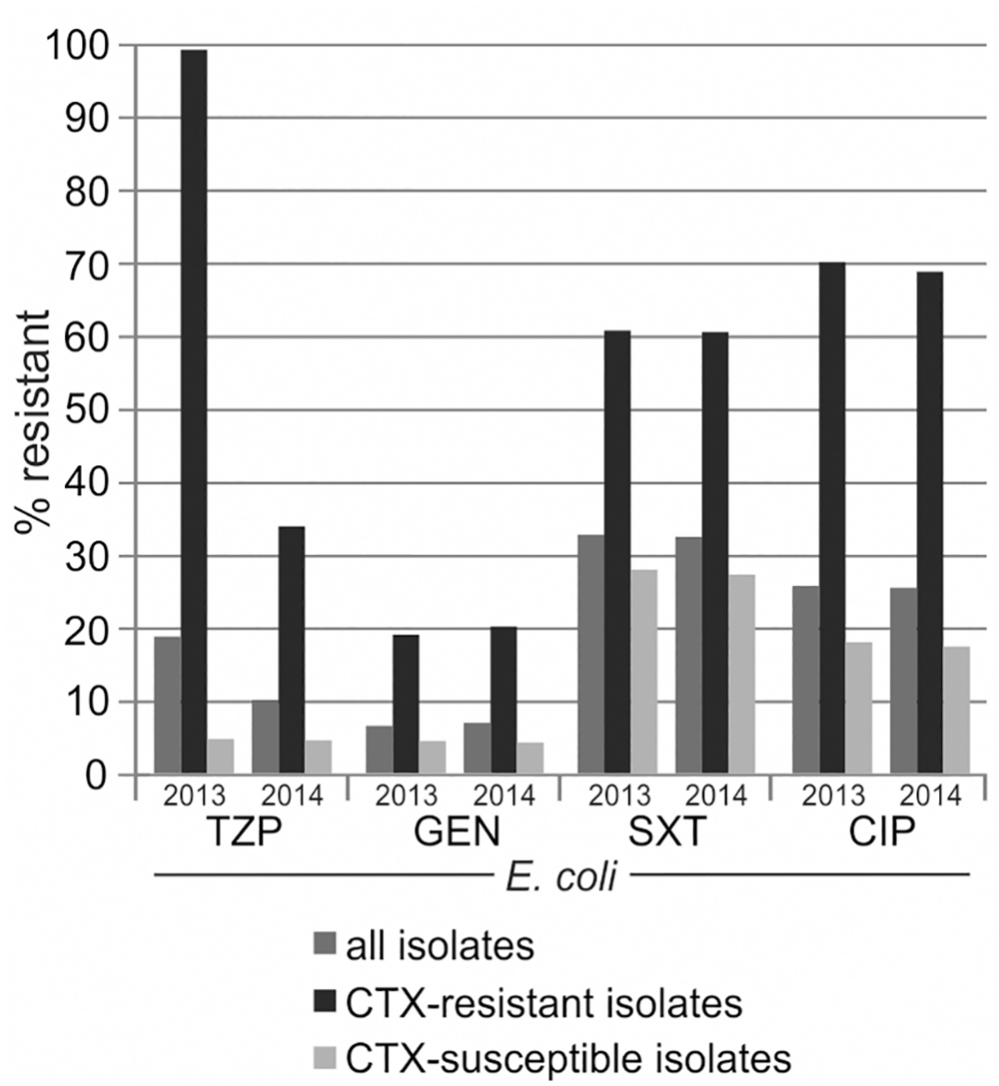
Resistance estimates dependent on organism’s resistance characteristics. Resistance rates were calculated with data stratification according to cefotaxime-resistance (all isolates: dark grey columns, only resistant strains: black columns, only susceptible strains: light grey columns), as described in the text. Further details are given in the supporting information (S6 Table).

We calculated cumulative antibiograms for selected species, either including all isolates of the given species or only those isolates with resistance or susceptibility to a first-line key antimicrobial (oxacillin in case of *Staphylococcus aureus* and cefotaxime in case of *Escherichia coli* and *Klebsiella pneumoniae*). For calculation, all isolates recovered in our laboratory were considered, after exclusion of screening isolates and duplicate isolate removal according to a 10 days episode strategy. Results are shown for *Escherichia coli* (Fig 6). Please refer to the supporting information for the number of included isolates and data on further selected species/antibiotic combinations (S6 Table). As expected, the oxacillin- or cefotaxime-resistant strains were more often resistant to further antimicrobials than the respective susceptible strains. For *Staphylococcus aureus*, differences were most pronounced for resistance to macrolides, lincosamides and fluoroquinolones (up to 81 percentage points). For *Escherichia coli* and *Klebsiella pneumoniae*, considerable differences in resistance rates were observed for piperacillin/tazobactam, ciprofloxacin, sulfamethoxazole/trimethoprim and gentamicin (up to 95, 65, 59 and 37 percentage points, respectively). However, resistance rates did not differ for fosfomycin and differences for tigecycline were found only in case of *Klebsiella pneumoniae*. Of note, the piperacillin/tazobactam-resistance rate of cefotaxime-resistant *Escherichia coli* and *Klebsiella pneumoniae* strains strikingly decreased from 2013 to 2014 because of changed interpretation rules for antimicrobial susceptibility testing (please refer to the chapter “Change in antimicrobial susceptibility testing methodology”).

Our data prove that resistance estimates may be biased when antimicrobials are tested only on a subset of isolates of a given species. In the case of second-line testing policies, higher resistance rates may be recorded for the supplementally tested antibiotics than would be if those agents were tested on all isolates. Thus, laboratories should either not report the results of second-line testing or generate cAST reports with stratification according to organism’s resistance characteristics. One previous study also identified second-line testing as the most influencing factor, with 7-13fold higher estimates of resistance compared to, e.g., up to 3fold higher estimates with prescribing-specific testing [34].

### Pooling of data from different species

We believe that cumulative antibiograms should be calculated at species level because of differences in susceptibility profiles for different species of one genus. However, several reports showed cAST data for the genus *Enterococcus* but not for the different *Enterococcus* species (e.g., [11, 12, 23, 26]).

Thus, we calculated cumulative antibiograms, considering either all enterococci isolates recovered in our laboratory or only the *Enterococcus faecalis* or *Enterococcus faecium* isolates. In each case, screening isolates were excluded and duplicate isolates removed according to a 10 days episode strategy. Our data prove considerable differences between genus-specific and species-specific resistance rates (e.g., ampicillin-resistance 28%, 0% and 90% in pooled enterococci, *Enterococcus faecalis* and *Enterococcus faecium*, respectively); for further details refer to the supporting information (S7 Table). Therefore, genus-specific resistance rates may be misleading.

Another example for pooling of data from different species is the direct testing and reporting of urinary coliform isolates (i.e., urine specimens are plated directly for antimicrobial susceptibility testing, without prior isolation and species identification). However, urinary tract isolates identified as *Escherichia coli* were shown to be significantly less resistant than those reported as undifferentiated coliforms [34].

### Change in antimicrobial susceptibility testing methodology

Comparability of cAST reports may be limited if a change in antimicrobial susceptibility testing methodology and interpretative reading of results occurs from one observation period to another, just as if different laboratories follow different procedures for antimicrobial susceptibility testing.

The change of antibiotic susceptibility testing guidelines from CLSI to EUCAST in 2011 was well-suited to demonstrate the influence of a method change on resistance estimates: When the same dataset was re-analyzed using the new breakpoints, resistance rates were up to 19 percentage points higher for several species/antibiotic combinations, with the most pronounced differences observed for Enterobacteriaceae [8, 30].

Here is another example for the effects that may be caused by a change in breakpoints for antimicrobial susceptibility testing: In 2014, the German NAC as the national branch of the EUCAST acknowledged that higher doses instead of the standard dose may be required for effective treatment of particular Enterobacteriaceae (e.g., *Escherichia coli*, *Klebsiella pneumoniae*, *Proteus mirabilis*) with certain antibiotics (aminopenicillins and 2^nd^ generation cephalosporins). Because this may be better reflected by reporting wild-type strains as intermediate instead of susceptible, susceptibility breakpoints were lowered (e.g., MIC breakpoint ≤0.5 mg/l instead of ≤8 mg/l for ampicillin). Our laboratory implemented the new breakpoints in March 2014. For comparison of resistance estimates before and after breakpoint change (2013 vs. April-December 2014), we considered all isolates recovered in our laboratory, after exclusion of screening and duplicate isolates as mentioned above. Because wild-type bacteria of the affected species were no longer considered susceptible but intermediate to ampicillin, ampicillin/sulbactam and cefuroxime, susceptibility rates of 0% were detected for the respective species/antibiotic combinations after implementation of the new breakpoints whereas non-resistance rates (susceptible + intermediate results) and resistance rates remained comparable; for further details refer to the supporting information (S8 Table). In contrast, susceptibility rates for cefotaxime were not affected. Thus, in laboratories following this recommendation, susceptibility rates are no longer feasible for monitoring of resistance trends. Possible solutions may be to report resistance rates, or to report intermediate results in addition to susceptibility rates with an explanatory note.

Similarly, the application of interpretation rules may profoundly influence resistance rates. Whereas some laboratories report every antimicrobial as tested, others perform rule-based interpretative reading of antimicrobial susceptibility testing results. However, rule-based susceptibility estimates may diverge markedly from test-based estimates, as was shown for erythromycin resistance in *Haemophilus influenzae* and ciprofloxacin resistance in Gram-positive cocci [34]. Another common rule-based reporting practice is to report all β-lactam antibiotics resistant apart from carbapenems for ESBL-producing Enterobacteriaceae, independent of the *in vitro* test result, to avoid *in vivo* treatment failure. This practice was also followed by our laboratory until we decided in the beginning of 2014 to report piperacillin/tazobactam intermediate instead of resistant for ESBL-producing Enterobacteriaceae in case of a MIC ≤4 mg/l, based on a study suggesting that high-dose piperacillin/tazobactam may be a suitable alternative to carbapenems for treating patients with infections due to ESBL-producing *Escherichia coli* [35]. This change in interpretation rules strikingly decreased piperacillin/tazobactam-resistance rates of cefotaxime-resistant *Escherichia coli* and *Klebsiella pneumoniae* strains from 100% to 40% and 70%, respectively, from 2013 to 2014 (compare Fig 6, S6 Table). As a result, lower overall piperacillin/tazobactam-resistance rates of Enterobacteriaceae were detected in 2014 compared to 2013 in all analyses shown in this study.

Of note, when modifications in antimicrobial susceptibility testing methodology and interpretation rules cause changes in cumulative antibiograms, clinicians may be misdirected in the interpretation of resistance trends in that true changes of resistance rates may be missed or unchanging resistance rates may be reported as increasing or decreasing. Thus, cAST data should always be analyzed in consideration of potential changes caused by amended antimicrobial susceptibility testing procedure.

## Conclusion

Several distinct approaches can be used for generation of cAST reports from a clinical microbiology laboratory database but, unfortunately, results obtained using different calculation algorithms have to be compared with caution. We have demonstrated the various influencing parameters that have to be considered when preparing or comparing cAST reports. Thus, national guidelines harmonizing cAST data analyses across laboratories are needed. Our opinion is that, in countries where such a document is not available, it is indispensable to have the construction methodologies notated on the cumulative antibiogram so that the calculation approach is transparent.

Of note, the resistance data presented in our study reflect local epidemiology and may not be generalizable to other geographic regions. In addition, local sampling practice, patient demographics and study setting may further hamper comparability of our results to those of others. We are, however, convinced that our data show that many parameters critically influence the resistance estimates.

Thus, we recommend that the parameters used during generation of cAST reports are communicated and explained to the clinicians. In particular, we suggest:

1. excluding isolates from surveillance and screening cultures,
2. discussing the effect of different duplicate isolate removal strategies with the served clinicians and, then, selecting one strategy dependent on the clinicians’ current needs,
3. calculating cumulative antibiograms annually and separately for each health care facility served by the laboratory, and discussing the various data stratification options with the served clinicians whereupon subanalyses may be performed dependent on the clinicians’ current needs and the number of isolates amenable to analysis,
4. avoiding selective testing policies and reporting resistance/susceptibility rates only for antibiotics routinely tested on all isolates,
5. calculating resistance/susceptibility rates at species level,
6. adhering to the EUCAST recommendations to ensure standardized antimicrobial susceptibility testing, and disclosing any change in antimicrobial susceptibility testing methodology and interpretative reading of results including its potential influence on future resistance estimates,
7. precisely defining and disclosing the calculation algorithm used for cAST report generation to facilitate comparability of cAST reports across different laboratories and observation periods.

## Supporting Information

S1 Table Resistance estimates dependent on the handling of screening isolates. Cumulative antibiograms were calculated either with inclusion or exclusion of screening isolates (all isolates vs. diagnostic isolates only), as detailed in the respective results and discussion section of the manuscript. In addition to the resistance rates for selected species/antibiotic combinations and the total number (n) of isolates included, the difference in resistance estimates between the different calculation approaches is shown (highlighted in light grey, with differences ≥5 percentage points in bold).
(PDF)

S2 Table Resistance estimates dependent on the method of duplicate isolate removal. Cumulative antibiograms were calculated using different methods of duplicate isolate removal, as detailed in the respective results and discussion section of the manuscript. The effect of the different duplicate isolate removal strategies is shown both on the total number of isolates included and on the resistance rates for selected species/antibiotic combinations. The percentage of isolates removed and the change in resistance estimates in comparison to the "all isolates" strategy are also shown (highlighted in light grey, with differences in resistance ≥5 percentage points in bold).
(PDF)

S3 Table Resistance estimates dependent on the time-point of isolate recovery. Cumulative antibiograms were calculated with respect to the time-point of isolate recovery in relation to the MRSA admission screening (early isolates: 1.-3. day; late isolates: ≥4. day), as detailed in the respective results and discussion section of the manuscript. In addition to the resistance rates for selected species/antibiotic combinations and the total number (n) of isolates included, the difference in resistance estimates between the different calculation approaches is shown (highlighted in light grey, with differences ≥5 percentage points in bold).
(PDF)

S4 Table Resistance estimates dependent on the patient location. Cumulative antibiograms were calculated with data stratification according to the patient location (hospital and ward), as detailed in the respective results and discussion section of the manuscript. In addition to the resistance rates for selected species/antibiotic combinations and the total number (n) of isolates included, the difference in resistance estimates between the different calculation approaches is shown (highlighted in light grey, with differences ≥5 percentage points in bold).
(PDF)

S5 Table Resistance estimates dependent on the specimen type. Cumulative antibiograms were calculated with data stratification according to the specimen type, as detailed in the respective results and discussion section of the manuscript. In addition to the resistance rates for selected species/antibiotic combinations and the total number (n) of isolates included, the difference in resistance estimates between the different calculation approaches is shown (highlighted in light grey, with differences ≥5 percentage points in bold).
(PDF)

S6 Table Resistance estimates dependent on organism’s resistance characteristics. Cumulative antibiograms were calculated with data stratification according to organism’s resistance characteristics regarding a first-line key antimicrobial (oxacillin for *Staphylococcus aureus* and cefotaxime for *Escherichia coli* and *Klebsiella pneumoniae*), as detailed in the respective results and discussion section of the manuscript. In addition to the resistance rates for selected species/antibiotic combinations and the total number (n) of isolates included, the difference in resistance estimates between the different calculation approaches is shown (highlighted in light grey, with differences ≥5 percentage points in bold).
(PDF)

S7 Table Genus-specific compared to species-specific resistance estimates. Cumulative antibiograms were calculated either at genus level or at species level, as detailed in the respective results and discussion section of the manuscript. In addition to the resistance rates for selected species/antibiotic combinations and the total number (n) of isolates included, the difference in resistance estimates between the different calculation approaches is shown (highlighted in light grey, with differences ≥5 percentage points in bold).
(PDF)

S8 Table Effect of a change in breakpoints for antimicrobial susceptibility testing. Susceptibility rates, non-resistance rates and resistance rates of *Escherichia coli*, *Klebsiella pneumoniae* and *Proteus mirabilis* isolates were calculated before (2013) and after change in breakpoints for antimicrobial susceptibility testing (Apr-Dec 2014), as detailed in the respective results and discussion section of the manuscript. The change in estimates from 2013 to Apr-Dec 2014 is also shown (highlighted in light grey, with differences resulting from the change in breakpoints in bold).
(PDF)
