# Supplementary material for: Analysis and Presentation of Cumulative Antimicrobial Susceptibility Test Data – The Influence of Different Parameters in a Routine Clinical Microbiology Laboratory
Source: PLoS One. 2016 Jan 27;11(1):e0147965. doi: 10.1371/journal.pone.0147965 (PMC4729434; doi:10.1371/journal.pone.0147965)
Supplement: S1 Table — Cumulative antibiograms were calculated either with inclusion or exclusion of screening isolates (all isolates vs. diagnostic isolates only), as detailed in the respective results and discussion section of the manuscript. In addition to the resistance rates for selected species/antibiotic combinations and the total number (n) of isolates included, the difference in resistance estimates between the different calculation approaches is shown (highlighted in light grey, with differences ≥5 percentage points in bold). (PDF) [file pone.0147965.s001.pdf]

S1 Table. Resistance estimates dependent on the handling of screening isolates.

Cumulative antibiograms were calculated either with inclusion or exclusion of screening isolates (all isolates vs. diagnostic isolates only), as detailed in the respective results and discussion section of the manuscript. In addition to the resistance rates for selected species/antibiotic combinations and the total number (n) of isolates included, the difference in resistance estimates between the different calculation approaches is shown (highlighted in light grey, with differences ≥5 percentage points in bold).

S. aureus		Resistance rate and difference in resistance estimates, respectively (in %)										
		PEN	OXA	GEN	ERY	CLI	SXT	TET	LVX	FOF	VAN	RIF
2013	all isolates, n=294	98.3	33.3	2.3	28.1	29.0	4.6	9.9	28.7	0.9	0.0	0.3
	diagnostic isolates only, n=222	97.7	13.6	1.9	15.1	15.1	3.4	5.7	15.1	0.8	0.0	0.4
	“all isolates” compared to “diagnostic isolates only”	+0.6	**+19.7**	+0.4	**+13.0**	**+13.9**	+1.2	+4.2	**+13.6**	+0.1	0.0	-0.1
2014	all isolates, n=307	99.3	33.7	2.3	28.7	28.3	3.3	8.1	27.8	0.7	0.0	0.0
	diagnostic isolates only, n=231	99.1	11.7	1.3	17.3	15.6	0.9	2.2	13.9	0.4	0.0	0.0
	“all isolates” compared to “diagnostic isolates only”	+0.2	**+22.0**	+1.0	**+11.4**	**+12.7**	+2.4	**+5.9**	**+13.9**	+0.3	0.0	0.0
