# Supplementary material for: Analysis and Presentation of Cumulative Antimicrobial Susceptibility Test Data – The Influence of Different Parameters in a Routine Clinical Microbiology Laboratory
Source: PLoS One. 2016 Jan 27;11(1):e0147965. doi: 10.1371/journal.pone.0147965 (PMC4729434; doi:10.1371/journal.pone.0147965)
Supplement: S2 Table — Cumulative antibiograms were calculated using different methods of duplicate isolate removal, as detailed in the respective results and discussion section of the manuscript. The effect of the different duplicate isolate removal strategies is shown both on the total number of isolates included and on the resistance rates for selected species/antibiotic combinations. The percentage of isolates removed and the change in resistance estimates in comparison to the "all isolates" strategy are also shown (highlighted in light grey, with differences in resistance ≥5 percentage points in bold). (PDF) [file pone.0147965.s002.pdf]

S2 Table. Resistance estimates dependent on the method of duplicate isolate removal.

Cumulative antibiograms were calculated using different methods of duplicate isolate removal, as detailed in the respective results and discussion section of the manuscript. The effect of the different duplicate isolate removal strategies is shown both on the total number of isolates included and on the resistance rates for selected species/antibiotic combinations. The percentage of isolates removed and the change in resistance estimates in comparison to the "all isolates" strategy are also shown (highlighted in light grey, with differences in resistance ≥5 percentage points in bold).

		Total number of isolates and percentage of isolates removed in comparison to "all isolates"									
		S. aureus		*E. coli*		*K. pneumoniae*		*P. aeruginosa*		*S. agalactiae*	
2013	all isolates	5966		9950		1932		2798		537	
	2 days episode	5513	-7.6%	9452	-5.0%	1825	-5.5%	2556	-8.6%	519	-3.4%
	5 days episode	5151	-13.7%	9105	-8.5%	1750	-9.4%	2344	-16.2%	512	-4.7%
	10 days episode	4899	-17.9%	8759	-12.0%	1647	-14.8%	2136	-23.7%	501	-6.7%
	30 days episode	4532	-24.0%	8105	-18.5%	1518	-21.4%	1834	-34.5%	485	-9.7%
	100 days episode	4109	-31.1%	7654	-23.1%	1436	-25.7%	1633	-41.6%	468	-12.8%
	first isolate	3919	-34.3%	7384	-25.8%	1403	-27.4%	1541	-44.9%	465	-13.4%
2014	all isolates	6072		12276		2216		3254		669	
	2 days episode	5641	-7.1%	11634	-5.2%	2116	-4.5%	2966	-8.9%	651	-2.7%
	5 days episode	5355	-11.8%	11191	-8.8%	2037	-8.1%	2761	-15.2%	642	-4.0%
	10 days episode	5092	-16.1%	10734	-12.6%	1955	-11.8%	2503	-23.1%	636	-4.9%
	30 days episode	4730	-22.1%	9988	-18.6%	1830	-17.4%	2199	-32.4%	624	-6.7%
	100 days episode	4238	-30.2%	9401	-23.4%	1727	-22.1%	1941	-40.4%	614	-8.2%
	first isolate	4035	-33.5%	9037	-26.4%	1686	-23.9%	1853	-43.1%	602	-10.0%

S. aureus		Resistance rate and difference in comparison to "all isolates" (in %)															
		PEN		OXA		GEN		ERY		CLI		TET		LVX		RIF	
2013	all isolates	97.9		30.8		2.6		33.9		32.6		4.4		37.9		0.9	
	2 days episode	98.0	+0.1	30.7	-0.1	2.7	+0.1	33.6	-0.3	32.4	-0.2	4.4	0.0	37.7	-0.2	0.8	-0.1
	5 days episode	97.9	0.0	30.3	-0.5	2.6	0.0	33.4	-0.5	32.2	-0.4	4.4	0.0	37.4	-0.5	0.8	-0.1
	10 days episode	97.9	0.0	30.2	-0.6	2.6	0.0	33.1	-0.8	32.0	-0.6	4.4	0.0	37.4	-0.5	0.8	-0.1
	30 days episode	97.8	-0.1	29.1	-1.7	2.6	0.0	31.8	-2.1	30.8	-1.8	4.4	0.0	36.0	-1.9	0.7	-0.2
	100 days episode	97.7	-0.2	27.5	-3.3	2.4	-0.2	30.1	-3.8	29.2	-3.4	4.5	+0.1	34.4	-3.5	0.6	-0.3
	first isolate	97.6	-0.3	26.9	-3.9	2.4	-0.2	29.5	-4.4	28.6	-4.0	4.5	+0.1	33.8	-4.1	0.5	-0.4
2014	all isolates	98.4		29.2		2.5		31.8		30.9		4.2		35.4		0.3	
	2 days episode	98.4	0.0	29.4	+0.2	2.6	+0.1	32.1	+0.3	31.1	+0.2	4.2	0.0	35.7	+0.3	0.4	+0.1
	5 days episode	98.3	-0.1	29.0	-0.2	2.6	+0.1	31.9	+0.1	31.0	+0.1	4.2	0.0	35.4	0.0	0.3	0.0
	10 days episode	98.3	-0.1	28.3	-0.9	2.6	+0.1	31.5	-0.3	30.7	-0.2	4.3	+0.1	34.8	-0.6	0.3	0.0
	30 days episode	98.3	-0.1	27.2	-2.0	2.5	0.0	30.5	-1.3	29.9	-1.0	4.2	0.0	33.6	-1.8	0.3	0.0
	100 days episode	98.1	-0.3	25.1	-4.1	2.5	0.0	28.3	-3.5	27.6	-3.3	4.0	-0.2	31.5	-3.9	0.2	-0.1
	first isolate	98.0	-0.4	24.6	-4.6	2.4	-0.1	27.9	-3.9	27.1	-3.8	3.9	-0.3	30.8	-4.6	0.2	-0.1

S2 Table continued.

E. coli		Resistance rate and difference in comparison to "all isolates" (in %)															
		AMP		SAM		TZP		CXM		CTX		GEN		SXT		CIP	
2013	all isolates	58.1		46.7		20.1		21.8		16.1		7.1		33.9		27.1	
	2 days episode	57.6	-0.5	46.0	-0.7	19.8	-0.3	21.5	-0.3	15.9	-0.2	7.0	-0.1	33.6	-0.3	26.6	-0.5
	5 days episode	57.2	-0.9	45.6	-1.1	19.4	-0.7	21.2	-0.6	15.6	-0.5	6.8	-0.3	33.2	-0.7	26.3	-0.8
	10 days episode	56.9	-1.2	45.1	-1.6	18.8	-1.3	20.7	-1.1	15.0	-1.1	6.6	-0.5	32.8	-1.1	25.8	-1.3
	30 days episode	55.9	-2.2	43.9	-2.8	17.7	-2.4	19.5	-2.3	14.2	-1.9	6.2	-0.9	31.9	-2.0	24.5	-2.6
	100 days episode	54.8	-3.3	42.7	-4.0	16.8	-3.3	18.6	-3.2	13.4	-2.7	5.9	-1.2	31.2	-2.7	23.9	-3.2
	first isolate	54.3	-3.8	42.1	-4.6	16.3	-3.8	18.0	-3.8	13.0	-3.1	5.8	-1.3	30.9	-3.0	23.2	-3.9
2014	all isolates	58.0		49.5		11.0		22.8		16.9		7.1		33.4		26.7	
	2 days episode	57.6	-0.4	48.2	-1.3	10.6	-0.4	22.4	-0.4	16.7	-0.2	6.9	-0.2	33.2	-0.2	26.5	-0.2
	5 days episode	57.1	-0.9	48.3	-1.2	10.3	-0.7	22.0	-0.8	16.2	-0.7	6.8	-0.3	32.9	-0.5	26.0	-0.7
	10 days episode	56.5	-1.5	47.6	-1.9	10.0	-1.0	21.4	-1.4	15.6	-1.3	6.6	-0.5	32.5	-0.9	25.5	-1.2
	30 days episode	55.3	-2.7	46.1	-3.4	9.3	-1.7	19.9	-2.9	14.3	-2.6	6.2	-0.9	31.4	-2.0	24.0	-2.7
	100 days episode	54.4	-3.6	45.1	-4.4	9.1	-1.9	19.0	-3.8	13.5	-3.4	6.0	-1.1	30.6	-2.8	23.0	-3.7
	first isolate	54.0	-4.0	44.9	-4.6	9.0	-2.0	18.5	-4.3	13.2	-3.7	5.9	-1.2	30.3	-3.1	22.4	-4.3
K. pneumoniae		Resistance rate and difference in comparison to "all isolates" (in %)															
		AMP		SAM		TZP		CXM		CTX		GEN		SXT		CIP	
2013	all isolates	100.0		34.1		23.2		25.7		18.7		6.8		18.3		15.8	
	2 days episode	100.0	0.0	33.2	-0.9	22.4	-0.8	25.1	-0.6	18.0	-0.7	6.3	-0.5	17.7	-0.6	15.3	-0.5
	5 days episode	100.0	0.0	32.1	-2.0	21.5	-1.7	24.5	-1.2	17.5	-1.2	6.0	-0.8	17.3	-1.0	15.2	-0.6
	10 days episode	100.0	0.0	31.0	-3.1	20.8	-2.4	23.4	-2.3	16.8	-1.9	5.8	-1.0	17.0	-1.3	14.7	-1.1
	30 days episode	100.0	0.0	29.3	-4.8	19.1	-4.1	21.5	-4.2	15.3	-3.4	5.4	-1.4	16.0	-2.3	13.6	-2.2
	100 days episode	100.0	0.0	28.6	**-5.5**	18.5	-4.7	20.8	-4.9	14.5	-4.2	5.3	-1.5	15.4	-2.9	12.9	-2.9
	first isolate	100.0	0.0	27.7	**-6.4**	17.6	**-5.6**	20.1	**-5.6**	13.7	**-5.0**	5.3	-1.5	15.0	-3.3	12.5	-3.3
2014	all isolates	100.0		27.8		13.0		21.5		13.0		6.0		14.8		11.5	
	2 days episode	100.0	0.0	27.5	-0.3	12.9	-0.1	21.3	-0.2	12.8	-0.2	5.9	-0.1	14.9	0.1	11.3	-0.2
	5 days episode	100.0	0.0	26.7	-1.1	12.8	-0.2	20.8	-0.7	12.5	-0.5	5.5	-0.5	14.3	-0.5	11.0	-0.5
	10 days episode	100.0	0.0	26.1	-1.7	12.6	-0.4	20.6	-0.9	12.2	-0.8	5.4	-0.6	14.2	-0.6	10.8	-0.7
	30 days episode	100.0	0.0	25.3	-2.5	12.0	-1.0	19.5	-2.0	11.9	-1.1	5.2	-0.8	13.9	-0.9	10.4	-1.1
	100 days episode	100.0	0.0	24.3	-3.5	11.4	-1.6	18.8	-2.7	11.1	-1.9	5.0	-1.0	12.9	-1.9	9.6	-1.9
	first isolate	100.0	0.0	24.3	-3.5	11.5	-1.5	18.8	-2.7	11.1	-1.9	5.0	-1.0	12.9	-1.9	9.7	-1.8

S2 Table continued.

P. aeruginosa		Resistance rate and difference in comparison to "all isolates" (in %)															
		TZP		CAZ		FEP		IPM		MEM		GEN		TOB		CIP	
2013	all isolates	29.2		18.3		15.4		26.4		9.2		16.2		8.3		21.6	
	2 days episode	28.4	-0.8	17.4	-0.9	14.6	-0.8	25.3	-1.1	8.2	-1.0	15.8	-0.4	7.7	-0.6	21.0	-0.6
	5 days episode	27.2	-2.0	16.6	-1.7	13.9	-1.5	23.2	-3.2	7.4	-1.8	15.6	-0.6	7.5	-0.8	20.3	-1.3
	10 days episode	24.5	-4.7	14.5	-3.8	12.4	-3.0	20.9	**-5.5**	6.5	-2.7	14.8	-1.4	7.1	-1.2	18.7	-2.9
	30 days episode	21.9	**-7.3**	11.8	**-6.5**	10.2	**-5.2**	18.6	**-7.8**	5.1	-4.1	13.1	-3.1	6.3	-2.0	16.9	-4.7
	100 days episode	19.2	**-10.0**	9.4	**-8.9**	8.0	**-7.4**	16.1	**-10.3**	4.0	**-5.2**	11.2	**-5.0**	5.2	-3.1	15.5	**-6.1**
	first isolate	19.8	**-9.4**	9.6	**-8.7**	8.2	**-7.2**	15.7	**-10.7**	3.8	**-5.4**	10.8	**-5.4**	5.2	-3.1	15.4	**-6.2**
2014	all isolates	24.6		16.4		12.8		22.8		9.4		11.2		6.9		22.0	
	2 days episode	23.8	-0.8	15.7	-0.7	12.4	-0.4	21.6	-1.2	8.7	-0.7	11.0	-0.2	6.4	-0.5	21.3	-0.7
	5 days episode	22.4	-2.2	14.7	-1.7	11.5	-1.3	20.1	-2.7	7.8	-1.6	10.5	-0.7	6.1	-0.8	20.3	-1.7
	10 days episode	20.4	-4.2	13.0	-3.4	10.2	-2.6	18.4	-4.4	7.2	-2.2	10.1	-1.1	5.8	-1.1	19.2	-2.8
	30 days episode	17.1	**-7.5**	10.7	**-5.7**	8.5	-4.3	15.7	**-7.1**	5.3	-4.1	9.3	-1.9	5.6	-1.3	17.0	**-5.0**
	100 days episode	14.9	**-9.7**	9.1	**-7.3**	7.3	**-5.5**	14.3	**-8.5**	4.5	-4.9	8.1	-3.1	5.0	-1.9	15.7	**-6.3**
	first isolate	14.6	**-10.0**	7.2	**-9.2**	7.2	**-5.6**	14.0	**-8.8**	4.4	**-5.0**	7.6	-3.6	4.7	-2.2	15.2	**-6.8**

S. agalactiae		Resistance rate and difference in comparison to "all isolates" (in %)									
		PEN		ERY		CLI		TET		MXF	
2013	all isolates	0.0		28.9		27.0		80.1		1.5	
	2 days episode	0.0	0.0	29.2	+0.3	27.1	+0.1	80.1	0.0	1.5	0.0
	5 days episode	0.0	0.0	29.5	+0.6	27.5	+0.5	80.1	0.0	1.5	0.0
	10 days episode	0.0	0.0	29.4	+0.5	27.3	+0.3	79.8	-0.3	1.4	-0.1
	30 days episode	0.0	0.0	29.2	+0.3	27.2	+0.2	80.2	+0.1	1.4	-0.1
	100 days episode	0.0	0.0	28.6	-0.3	26.7	-0.3	79.9	-0.2	1.5	0.0
	first isolate	0.0	0.0	28.6	-0.3	26.7	-0.3	79.7	-0.4	1.5	0.0
2014	all isolates	0.0		29.4		24.6		77.5		2.5	
	2 days episode	0.0	0.0	29.2	-0.2	24.4	-0.2	77.9	+0.4	2.4	-0.1
	5 days episode	0.0	0.0	29.3	-0.1	24.6	0.0	78.0	+0.5	2.4	-0.1
	10 days episode	0.0	0.0	29.0	-0.4	24.2	-0.4	78.0	+0.5	2.5	0.0
	30 days episode	0.0	0.0	28.7	-0.7	23.9	-0.7	78.0	+0.5	2.5	0.0
	100 days episode	0.0	0.0	28.4	-1.0	23.8	-0.8	78.0	+0.5	2.6	+0.1
	first isolate	0.0	0.0	28.5	-0.9	23.9	-0.7	78.0	+0.5	2.6	+0.1
