# Supplementary material for: Analysis and Presentation of Cumulative Antimicrobial Susceptibility Test Data – The Influence of Different Parameters in a Routine Clinical Microbiology Laboratory
Source: PLoS One. 2016 Jan 27;11(1):e0147965. doi: 10.1371/journal.pone.0147965 (PMC4729434; doi:10.1371/journal.pone.0147965)
Supplement: S3 Table — Cumulative antibiograms were calculated with respect to the time-point of isolate recovery in relation to the MRSA admission screening (early isolates: 1.-3. day; late isolates: ≥4. day), as detailed in the respective results and discussion section of the manuscript. In addition to the resistance rates for selected species/antibiotic combinations and the total number (n) of isolates included, the difference in resistance estimates between the different calculation approaches is shown (highlighted in light grey, with differences ≥5 percentage points in bold). (PDF) [file pone.0147965.s003.pdf]

S3 Table. Resistance estimates dependent on the time-point of isolate recovery.

Cumulative antibiograms were calculated with respect to the time-point of isolate recovery in relation to the MRSA admission screening (early isolates: 1.-3. day; late isolates: ≥4. day), as detailed in the respective results and discussion section of the manuscript. In addition to the resistance rates for selected species/antibiotic combinations and the total number (n) of isolates included, the difference in resistance estimates between the different calculation approaches is shown (highlighted in light grey, with differences ≥5 percentage points in bold).

S. aureus		Resistance rate and difference in resistance estimates, respectively (in %)							
		PEN	OXA	GEN	ERY	CLI	TET	LVX	RIF
2013	early isolates, n=2030	97.5	28.7	2.8	32.0	30.7	4.1	37.5	0.6
	late isolates, n=1586	98.1	36.1	2.9	39.0	38.2	4.2	45.0	0.8
	"late isolates" compared to "early isolates"	+0.6	**+7.4**	+0.1	**+7.0**	**+7.5**	+0.1	**+7.5**	+0.2
2014	early isolates, n=2137	97.7	25.5	2.7	29.6	28.9	4.0	34.5	0.2
	late isolates, n=1958	98.3	32.4	2.4	35.0	34.4	3.4	40.7	0.2
	"late isolates" compared to "early isolates"	+0.6	**+6.9**	-0.3	**+5.4**	**+5.5**	-0.6	**+6.2**	0.0
E. coli		Resistance rate and difference in resistance estimates, respectively (in %)							
		AMP	SAM	TZP	CXM	CTX	GEN	SXT	CIP
2013	early isolates, n=3848	56.1	43.8	17.5	19.2	14.0	6.4	31.5	25.3
	late isolates, n=2923	60.4	47.3	20.8	22.1	16.7	7.1	34.2	28.9
	"late isolates" compared to "early isolates"	+4.3	+3.5	+3.3	+2.9	+2.7	+0.7	+2.7	+3.6
2014	early isolates, n=4892	54.1	45.0	9.3	18.5	12.8	5.6	30.5	23.6
	late isolates, n=4190	58.2	49.1	11.3	22.1	15.8	6.4	33.0	27.2
	"late isolates" compared to "early isolates"	+4.1	+4.1	+2.0	+3.6	+3.0	+0.8	+2.5	+3.6
K. pneumoniae		Resistance rate and difference in resistance estimates, respectively (in %)							
		AMP	SAM	TZP	CXM	CTX	GEN	SXT	CIP
2013	early isolates, n=754	99.7	29.4	17.8	21.1	14.5	5.6	15.0	12.6
	late isolates, n=614	100.0	30.5	19.7	22.8	16.0	6.0	16.2	15.5
	"late isolates" compared to "early isolates"	+0.3	+1.1	+1.9	+1.7	+1.5	+0.4	+1.2	+2.9
2014	early isolates, n=961	100.0	22.7	11.2	17.9	10.8	5.1	12.1	8.7
	late isolates, n=920	100.0	27.3	12.5	21.1	13.5	5.4	14.2	10.5
	"late isolates" compared to "early isolates"	0.0	+4.6	+1.3	+3.2	+2.7	+0.3	+2.1	+1.8
P. aeruginosa		Resistance rate and difference in resistance estimates, respectively (in %)							
		TZP	CAZ	FEP	IPM	MEM	GEN	TOB	CIP
2013	early isolates, n=897	20.7	9.8	8.5	16.9	3.9	11.1	5.1	15.6
	late isolates, n=752	22.8	12.8	10.1	17.7	4.7	11.3	5.5	16.3
	"late isolates" compared to "early isolates"	+2.1	+3.0	+1.6	+0.8	+0.8	+0.2	+0.4	+0.7
2014	early isolates, n=1063	16.7	9.9	7.2	15.7	4.3	8.0	4.4	16.1
	late isolates, n=1117	17.3	11.5	8.5	15.0	4.9	8.0	5.0	16.3
	"late isolates" compared to "early isolates"	+0.6	+1.6	+1.3	-0.7	+0.6	0.0	+0.6	+0.2

S3 Table continued.

S. agalactiae		Resistance rate and difference in resistance estimates, respectively (in %)				
		PEN	ERY	CLI	TET	MXF
2013	early isolates, n=205	0.0	28.3	26.4	81.7	1.9
	late isolates, n=110	0.0	29.2	24.8	82.5	1.8
	“late isolates” compared to “early isolates”	0.0	+0.9	-1.6	+0.8	-0.1
2014	early isolates, n=290	0.3	26.4	21.1	76.3	2.0
	late isolates, n=176	0.6	30.0	24.3	73.9	2.0
	“late isolates” compared to “early isolates”	0.3	+3.6	+3.2	-2.4	0.0
