# Supplementary material for: Analysis and Presentation of Cumulative Antimicrobial Susceptibility Test Data – The Influence of Different Parameters in a Routine Clinical Microbiology Laboratory
Source: PLoS One. 2016 Jan 27;11(1):e0147965. doi: 10.1371/journal.pone.0147965 (PMC4729434; doi:10.1371/journal.pone.0147965)
Supplement: S4 Table — Cumulative antibiograms were calculated with data stratification according to the patient location (hospital and ward), as detailed in the respective results and discussion section of the manuscript. In addition to the resistance rates for selected species/antibiotic combinations and the total number (n) of isolates included, the difference in resistance estimates between the different calculation approaches is shown (highlighted in light grey, with differences ≥5 percentage points in bold). (PDF) [file pone.0147965.s004.pdf]

S4 Table. Resistance estimates dependent on the patient location.

Cumulative antibiograms were calculated with data stratification according to the patient location (hospital and ward), as detailed in the respective results and discussion section of the manuscript. In addition to the resistance rates for selected species/antibiotic combinations and the total number (n) of isolates included, the difference in resistance estimates between the different calculation approaches is shown (highlighted in light grey, with differences ≥5 percentage points in bold).

S. aureus		Resistance rate and difference in resistance estimates, respectively (in %)								
		PEN	OXA	GEN	ERY	CLI	TET	LVX	FOF	RIF
2013	Hospital A – ICU, n=108	99.1	41.7	1.9	38.0	38.3	5.6	50.9	0.0	0.9
	Hospital A – non-ICU, n=1111	97.3	24.7	3.1	33.8	32.3	4.2	31.8	1.0	0.7
	Hospital B – ICU, n=62	100.0	32.3	8.1	41.9	37.1	4.8	38.7	4.8	1.6
	Hospital B – non-ICU, n=609	98.2	35.5	3.1	31.0	30.5	3.6	41.0	2.3	0.8
	"A – ICU" compared to "A – non-ICU"	+1.8	**+17.0**	-1.2	+4.2	**+6.0**	+1.4	**+19.1**	-1.0	+0.2
	"B – ICU" compared to "B – non-ICU"	+1.8	-3.2	**+5.0**	**+10.9**	**+6.6**	+1.2	-2.3	+2.5	+0.8
	"A – ICU" compared to "B – ICU"	-0.9	**+9.4**	**-6.2**	-3.9	+1.2	+0.8	**+12.2**	-4.8	-0.7
	"A – non-ICU" compared to "B – non-ICU"	-0.9	**-10.8**	0.0	+2.8	+1.8	+0.6	**-9.2**	-1.3	-0.1
2014	Hospital A – ICU, n=103	98.1	32.7	1.9	35.6	35.9	4.8	38.5	0.0	1.0
	Hospital A – non-ICU, n=1216	98.6	24.1	3.7	29.6	27.8	4.5	28.0	1.4	0.5
	Hospital B – ICU, n=60	95.0	18.3	0.0	16.7	13.3	3.3	22.4	1.7	0.0
	Hospital B – non-ICU, n=568	98.1	32.0	1.4	34.5	33.6	3.9	39.5	1.8	0.5
	"A – ICU" compared to "A – non-ICU"	-0.5	**+8.6**	-1.8	**+6.0**	**+8.1**	+0.3	**+10.5**	-1.4	+0.5
	"B – ICU" compared to "B – non-ICU"	-3.1	**-13.7**	-1.4	**-17.8**	**-20.3**	-0.6	**-17.1**	-0.1	-0.5
	"A – ICU" compared to "B – ICU"	+3.1	**+14.4**	+1.9	**+18.9**	**+22.6**	+1.5	**+16.1**	-1.7	+1.0
	"A – non-ICU" compared to "B – non-ICU"	+0.5	**-7.9**	+2.3	-4.9	**-5.8**	+0.6	**-11.5**	-0.4	0.0
E. coli		Resistance rate and difference in resistance estimates, respectively (in %)								
		AMP	SAM	TZP	CXM	CTX	IPM	GEN	SXT	CIP
2013	Hospital A – ICU, n=134	58.2	46.3	20.1	23.1	13.4	0.0	7.5	31.3	26.1
	Hospital A – non-ICU, n=1121	58.3	47.5	19.6	23.2	17.0	0.0	5.9	33.0	25.6
	Hospital B – ICU, n=88	73.9	62.5	31.8	36.4	26.1	0.0	13.6	44.8	29.5
	Hospital B – non-ICU, n=1877	56.8	44.2	17.9	21.2	15.3	0.0	6.1	32.9	28.0
	"A – ICU" compared to "A – non-ICU"	-0.1	-1.2	+0.5	-0.1	-3.6	0.0	+1.6	-1.7	+0.5
	"B – ICU" compared to "B – non-ICU"	**+17.1**	**+18.3**	**+13.9**	**+15.2**	**+10.8**	0.0	**+7.5**	**+11.9**	+1.5
	"A – ICU" compared to "B – ICU"	**-15.7**	**-16.2**	**-11.7**	**-13.3**	**-12.7**	0.0	**-6.1**	**-13.5**	-3.4
	"A – non-ICU" compared to "B – non-ICU"	+1.5	+3.3	+1.7	+2.0	+1.7	0.0	-0.2	+0.1	-2.4
2014	Hospital A – ICU, n=175	55.4	45.7	14.9	28.6	20.0	0.0	8.0	32.0	33.7
	Hospital A – non-ICU, n=1287	55.5	46.9	10.4	22.9	14.5	0.0	6.3	35.6	27.0
	Hospital B – ICU, n=134	66.4	60.8	18.7	41.8	37.3	0.0	9.7	34.3	26.9
	Hospital B – non-ICU, n=1985	57.7	49.4	10.1	23.2	16.7	0.0	7.9	32.5	29.5
	"A – ICU" compared to "A – non-ICU"	-0.1	-1.2	+4.5	**+5.7**	**+5.5**	0.0	+1.7	-3.6	**+6.7**
	"B – ICU" compared to "B – non-ICU"	**+8.7**	**+11.4**	**+8.6**	**+18.6**	**+20.6**	0.0	+1.8	+1.8	-2.6
	"A – ICU" compared to "B – ICU"	**-11.0**	**-15.1**	-3.8	**-13.2**	**-17.3**	0.0	-1.7	-2.3	**+6.8**
	"A – non-ICU" compared to "B – non-ICU"	-2.2	-2.5	+0.3	-0.3	-2.2	0.0	-1.6	+3.1	-2.5

S4 Table continued.

K. pneumoniae		Resistance rate and difference in resistance estimates, respectively (in %)								
		AMP	SAM	TZP	CXM	CTX	IPM	GEN	SXT	CIP
2013	Hospital A – ICU, n=46	100.0	32.6	24.4	32.6	19.6	0.0	4.3	15.6	19.6
	Hospital A – non-ICU, n=241	100.0	27.0	15.8	19.5	10.8	0.0	5.0	12.0	9.1
	Hospital B – ICU, n=26	100.0	19.2	15.4	15.4	15.4	0.0	15.4	19.2	23.1
	Hospital B – non-ICU, n=372	100.0	35.2	26.9	29.3	23.1	0.0	9.4	17.2	20.4
	"A – ICU" compared to "A – non-ICU"	0.0	**+5.6**	**+8.6**	**+13.1**	**+8.8**	0.0	-0.7	+3.6	**+10.5**
	"B – ICU" compared to "B – non-ICU"	0.0	**-16.0**	**-11.5**	**-13.9**	**-7.7**	0.0	**+6.0**	+2.0	+2.7
	"A – ICU" compared to "B – ICU"	0.0	**+13.4**	**+9.0**	**+17.2**	+4.2	0.0	**-11.1**	-3.6	-3.5
	"A – non-ICU" compared to "B – non-ICU"	0.0	**-8.2**	**-11.1**	**-9.8**	**-12.3**	0.0	-4.4	**-5.2**	**-11.3**
2014	Hospital A – ICU, n=50	100.0	36.0	14.0	40.0	16.0	0.0	0.0	16.0	10.0
	Hospital A – non-ICU, n=267	100.0	26.2	12.0	18.0	10.2	0.0	6.0	11.3	9.4
	Hospital B – ICU, n=39	100.0	25.6	2.6	23.1	7.7	0.0	5.1	5.1	5.1
	Hospital B – non-ICU, n=361	100.0	34.2	17.5	29.0	18.0	0.0	9.9	20.2	18.2
	"A – ICU" compared to "A – non-ICU"	0.0	**+9.8**	+2.0	**+22.0**	**+5.8**	0.0	**-6.0**	**+4.7**	+0.6
	"B – ICU" compared to "B – non-ICU"	0.0	**-8.6**	**-14.9**	**-5.9**	**-10.3**	0.0	-4.8	**-15.1**	**-13.1**
	"A – ICU" compared to "B – ICU"	0.0	**+10.4**	**+11.4**	**+16.9**	**+8.3**	0.0	**-5.1**	**+10.9**	+4.9
	"A – non-ICU" compared to "B – non-ICU"	0.0	**-8.0**	**-5.5**	**-11.0**	**-7.8**	0.0	-3.9	**-8.9**	**-8.8**
P. aeruginosa		Resistance rate and difference in resistance estimates, respectively (in %)								
		PIP	TZP	CAZ	FEP	IPM	MEM	GEN	TOB	CIP
2013	Hospital A – ICU, n=65	40.0	40.0	33.3	24.6	40.0	13.6	27.3	18.5	40.9
	Hospital A – non-ICU, n=452	37.2	33.8	22.4	19.1	26.0	13.7	25.1	10.3	19.3
	Hospital B – ICU, n=39	33.3	28.2	15.4	20.5	30.8	12.8	30.8	30.8	35.9
	Hospital B – non-ICU, n=313	18.5	15.5	9.7	8.3	10.1	1.9	7.9	3.6	17.9
	"A – ICU" compared to "A – non-ICU"	+2.8	**+6.2**	**+10.9**	**+5.5**	**+14.0**	-0.1	+2.2	**+8.2**	**+21.6**
	"B – ICU" compared to "B – non-ICU"	**+14.8**	**+12.7**	**+5.7**	**+12.2**	**+20.7**	**+10.9**	**+22.9**	**+27.2**	**+18.0**
	"A – ICU" compared to "B – ICU"	**+6.7**	**+11.8**	**+17.9**	+4.1	**+9.2**	+0.8	-3.5	**-12.3**	**+5.0**
	"A – non-ICU" compared to "B – non-ICU"	**+18.7**	**+18.3**	**+12.7**	**+10.8**	**+15.9**	**+11.8**	**+17.2**	**+6.7**	+1.4
2014	Hospital A – ICU, n=87	34.5	32.2	23.9	16.5	38.6	14.8	17.0	11.6	30.7
	Hospital A – non-ICU, n=515	28.9	25.4	17.1	14.4	27.4	12.2	16.4	9.2	20.4
	Hospital B – ICU, n=46	37.0	36.2	21.3	18.2	37.0	21.3	15.2	13.6	25.5
	Hospital B – non-ICU, n=316	14.9	10.8	7.2	6.8	9.6	3.9	7.2	4.5	16.5
	"A – ICU" compared to "A – non-ICU"	**+5.6**	**+6.8**	**+6.8**	+2.1	**+11.2**	+2.6	+0.6	+2.4	**+10.3**
	"B – ICU" compared to "B – non-ICU"	**+22.1**	**+25.4**	**+14.1**	**+11.4**	**+27.4**	**+17.4**	**+8.0**	**+9.1**	**+9.0**
	"A – ICU" compared to "B – ICU"	-2.5	-4.0	2.6	-1.7	+1.6	**-6.5**	+1.8	-2.0	**+5.2**
	"A – non-ICU" compared to "B – non-ICU"	**+14.0**	**+14.6**	**+9.9**	**+7.6**	**+17.8**	**+8.3**	**+9.2**	+4.7	+3.9
