# Supplementary material for: Analysis and Presentation of Cumulative Antimicrobial Susceptibility Test Data – The Influence of Different Parameters in a Routine Clinical Microbiology Laboratory
Source: PLoS One. 2016 Jan 27;11(1):e0147965. doi: 10.1371/journal.pone.0147965 (PMC4729434; doi:10.1371/journal.pone.0147965)
Supplement: S5 Table — Cumulative antibiograms were calculated with data stratification according to the specimen type, as detailed in the respective results and discussion section of the manuscript. In addition to the resistance rates for selected species/antibiotic combinations and the total number (n) of isolates included, the difference in resistance estimates between the different calculation approaches is shown (highlighted in light grey, with differences ≥5 percentage points in bold). (PDF) [file pone.0147965.s005.pdf]

S5 Table. Resistance estimates dependent on the specimen type.

Cumulative antibiograms were calculated with data stratification according to the specimen type, as detailed in the respective results and discussion section of the manuscript. In addition to the resistance rates for selected species/antibiotic combinations and the total number (n) of isolates included, the difference in resistance estimates between the different calculation approaches is shown (highlighted in light grey, with differences ≥5 percentage points in bold).

S. aureus		Resistance rate and difference in resistance estimates, respectively (in %)								
		PEN	OXA	GEN	ERY	CLI	TET	LVX	FOF	RIF
2013	Blood culture isolates, n=416	96.4	23.0	2.6	31.2	30.5	2.2	33.8	1.4	0.7
	Non-blood culture isolates, n=4660	97.9	30.9	2.6	33.4	32.3	4.6	37.8	1.3	0.8
	"Blood culture isolates" compared to "Non-blood culture isolates"	-1.5	**-7.9**	0.0	-2.2	-1.8	-2.4	-4.0	+0.1	-0.1
2014	Blood culture isolates, n=438	98.6	16.9	0.9	22.8	22.1	2.1	24.8	0.9	0.9
	Non-blood culture isolates, n=4835	98.3	29.2	2.7	32.1	32.1	4.4	35.5	1.2	0.3
	"Blood culture isolates" compared to "Non-blood culture isolates"	+0.3	**-12.3**	-1.8	**-9.3**	**-10.0**	-2.3	**-10.7**	-0.3	+0.6
E. coli		Resistance rate and difference in resistance estimates, respectively (in %)								
		AMP	SAM	TZP	CXM	CTX	IPM	GEN	SXT	CIP
2013	Blood culture isolates, n=554	64.1	53.2	20.0	21.6	16.8	0.2	9.4	38.6	33.9
	Non-blood culture isolates, n=8398	56.5	44.8	18.8	20.6	15.0	0.0	6.4	32.6	25.4
	"Blood culture isolates" compared to "Non-blood culture isolates"	**+7.6**	**+8.4**	+1.2	+1.0	+1.8	+0.2	+3.0	**+6.0**	**+8.5**
2014	Blood culture isolates, n=686	54.1	50.4	9.9	18.2	13.7	0.0	6.9	32.7	23.4
	Non-blood culture isolates, n=10211	56.5	47.2	10.1	21.0	15.2	0.0	6.7	32.3	25.3
	"Blood culture isolates" compared to "Non-blood culture isolates"	-2.4	+3.2	-0.2	-2.8	-1.5	0.0	+0.2	+0.4	-1.9
K. pneumoniae		Resistance rate and difference in resistance estimates, respectively (in %)								
		AMP	SAM	TZP	CXM	CTX	IPM	GEN	SXT	CIP
2013	Blood culture isolates, n=108	100.0	31.5	21.3	21.8	13.0	0.0	6.5	18.5	10.2
	Non-blood culture isolates, n=1573	100.0	31.4	21.3	24.1	17.3	0.1	6.0	17.2	15.3
	"Blood culture isolates" compared to "Non-blood culture isolates"	0.0	+0.1	0.0	-2.3	-4.3	-0.1	+0.5	+1.3	**-5.1**
2014	Blood culture isolates, n=127	100.0	31.7	15.7	21.3	15.0	0.0	7.1	13.5	11.0
	Non-blood culture isolates, n=1876	100.0	26.2	12.7	20.9	12.3	0.0	5.3	14.3	10.8
	"Blood culture isolates" compared to "Non-blood culture isolates"	0.0	**+5.5**	+3.0	+0.4	+2.7	0.0	+1.8	-0.8	+0.2
P. aeruginosa		Resistance rate and difference in resistance estimates, respectively (in %)								
		PIP	TZP	CAZ	FEP	IPM	MEM	GEN	TOB	CIP
2013	Blood culture isolates, n=53	13.2	15.1	9.6	6.4	15.1	5.7	7.8	0.0	7.5
	Non-blood culture isolates, n=2106	27.7	24.7	14.5	12.4	21.1	6.6	14.9	7.2	18.9
	"Blood culture isolates" compared to "Non-blood culture isolates"	**-14.5**	**-9.6**	-4.9	**-6.0**	**-6.0**	-0.9	**-7.1**	**-7.2**	**-11.4**
2014	Blood culture isolates, n=79	21.5	20.5	13.9	11.4	13.9	7.6	10.1	5.1	16.5
	Non-blood culture isolates, n=2457	23.9	20.6	13.1	10.3	18.5	7.2	10.1	5.9	19.4
	"Blood culture isolates" compared to "Non-blood culture isolates"	-2.4	-0.1	+0.8	+1.1	-4.6	+0.4	0.0	-0.8	-2.9
