# Supplementary material for: Analysis and Presentation of Cumulative Antimicrobial Susceptibility Test Data – The Influence of Different Parameters in a Routine Clinical Microbiology Laboratory
Source: PLoS One. 2016 Jan 27;11(1):e0147965. doi: 10.1371/journal.pone.0147965 (PMC4729434; doi:10.1371/journal.pone.0147965)
Supplement: S6 Table — Cumulative antibiograms were calculated with data stratification according to organism’s resistance characteristics regarding a first-line key antimicrobial (oxacillin for Staphylococcus aureus and cefotaxime for Escherichia coli and Klebsiella pneumoniae), as detailed in the respective results and discussion section of the manuscript. In addition to the resistance rates for selected species/antibiotic combinations and the total number (n) of isolates included, the difference in resistance estimates between the different calculation approaches is shown (highlighted in light grey, with differences ≥5 percentage points in bold). (PDF) [file pone.0147965.s006.pdf]

S6 Table. Resistance estimates dependent on organism's resistance characteristics.

Cumulative antibiograms were calculated with data stratification according to organism's resistance characteristics regarding a first-line key antimicrobial (oxacillin for *Staphylococcus aureus* and cefotaxime for *Escherichia coli* and *Klebsiella pneumoniae*), as detailed in the respective results and discussion section of the manuscript. In addition to the resistance rates for selected species/antibiotic combinations and the total number (n) of isolates included, the difference in resistance estimates between the different calculation approaches is shown (highlighted in light grey, with differences ≥5 percentage points in bold).

S. aureus		Resistance rate and difference in resistance estimates, respectively (in %)							
		OXA	GEN	ERY	CLI	LVX	FOF	VAN	RIF
2013	All isolates, n=4897	30.5	2.7	33.2	32.2	37.9	1.3	0.0	0.8
	OXA-resistant isolates, n=1514	100.0	3.9	76.3	75.9	94.1	2.8	0.0	2.0
	OXA-susceptible isolates, n=3451	0.0	2.0	14.3	13.0	13.4	0.6	0.0	0.3
	"OXA-resistant isolates" compared to "all isolates"	**+69.5**	+1.2	**+43.1**	**+43.7**	**+56.2**	+1.5	0.0	+1.2
	"OXA-resistant isolates" compared to "OXA-susceptible isolates"	**+100.0**	+1.9	**+62.0**	**+62.9**	**+80.7**	+2.2	0.0	+1.7
	"OXA-susceptible isolates" compared to "all isolates"	**-30.5**	-0.7	**-18.9**	**-19.2**	**-24.5**	-0.7	0.0	-0.5
2014	All isolates, n=4989	27.9	2.6	31.2	30.3	34.5	1.2	0.0	0.3
	OXA-resistant isolates, n=1409	100.0	3.9	71.8	71.5	91.6	2.2	0.1	0.8
	OXA-susceptible isolates, n=3618	0.0	2.1	15.6	14.5	12.6	0.8	0.0	0.2
	"OXA-resistant isolates" compared to "all isolates"	**+72.1**	+1.3	**+40.6**	**+41.2**	**+57.1**	+1.0	+0.1	+0.5
	"OXA-resistant isolates" compared to "OXA-susceptible isolates"	**+100.0**	+1.8	**+56.2**	**+57.0**	**+79.0**	+1.4	+0.1	+0.6
	"OXA-susceptible isolates" compared to "all isolates"	**-27.9**	-0.5	**-15.6**	**-15.8**	**-21.9**	-0.4	0.0	-0.1
E. coli		Resistance rate and difference in resistance estimates, respectively (in %)							
		CTX	TZP	IPM	GEN	SXT	TGC	CIP	FOF
2013	All isolates, n=8754	15.0	18.7	0.0	6.5	32.8	0.6	25.8	1.9
	CTX-resistant isolates, n=1342	100.0	100.0	0.0	18.9	60.8	0.6	70.2	2.6
	CTX-susceptible isolates, n=7469	0.0	4.7	0.0	4.4	28.0	0.6	18.0	1.7
	"CTX-resistant isolates" compared to "all isolates"	**+85.0**	**+81.3**	0.0	**+12.4**	**+28.0**	0.0	**+44.4**	+0.7
	"CTX-resistant isolates" compared to "CTX-susceptible isolates"	**+100.0**	**+95.3**	0.0	**+14.5**	**+32.8**	0.0	**+52.2**	+0.9
	"CTX-susceptible isolates" compared to "all isolates"	**-15.0**	**-14.0**	0.0	-2.1	-4.8	0.0	**-7.8**	-0.2
2014	All isolates, n=10736	15.6	10.0	0.0	6.9	32.5	0.5	25.5	1.5
	CTX-resistant isolates, n=1726	100.0	39.7	0.1	20.0	60.6	0.4	68.9	2.7
	CTX-susceptible isolates, n=9113	0.0	4.5	0.0	4.2	27.3	0.5	17.4	1.3
	"CTX-resistant isolates" compared to "all isolates"	**+84.4**	**+29.7**	+0.1	**+13.1**	**+28.1**	-0.1	**+43.4**	+1.2
	"CTX-resistant isolates" compared to "CTX-susceptible isolates"	**+100.0**	**+35.2**	+0.1	**+15.8**	**+33.3**	-0.1	**+51.5**	+1.4
	"CTX-susceptible isolates" compared to "all isolates"	**-15.6**	**-5.5**	0.0	-2.7	**-5.2**	0.0	**-8.1**	-0.2

S6 Table continued.

K. pneumoniae		Resistance rate and difference in resistance estimates, respectively (in %)							
		CTX	TZP	IPM	GEN	SXT	TGC	CIP	FOF
2013	All isolates, n=1644	16.8	20.8	0.1	5.8	16.9	10.8	14.8	18.8
	CTX-resistant isolates, n=283	100.0	100.0	0.4	27.6	66.0	21.9	68.9	19.9
	CTX-susceptible isolates, n=1373	0.0	4.6	0.0	1.5	7.1	8.6	3.7	18.7
	"CTX-resistant isolates" compared to "all isolates"	**+83.2**	**+79.2**	+0.3	**+21.8**	**+49.1**	**+11.1**	**+54.1**	+1.1
	"CTX-resistant isolates" compared to "CTX-susceptible isolates"	**+100.0**	**+95.4**	+0.4	**+26.1**	**+58.9**	**+13.3**	**+65.2**	+1.2
	"CTX-susceptible isolates" compared to "all isolates"	**-16.8**	**-16.2**	-0.1	-4.3	**-9.8**	-2.2	**-11.1**	-0.1
2014	All isolates, n=1956	12.2	12.6	0.0	5.4	14.3	11.6	10.8	21.9
	CTX-resistant isolates, n=243	100.0	70.4	0.0	37.7	63.8	15.8	54.1	21.2
	CTX-susceptible isolates, n=1718	0.0	4.4	0.0	0.8	7.3	10.9	4.9	22.2
	"CTX-resistant isolates" compared to "all isolates"	**+87.8**	**+57.8**	0.0	**+32.3**	**+49.5**	+4.2	**+43.3**	-0.7
	"CTX-resistant isolates" compared to "CTX-susceptible isolates"	**+100.0**	**+66.0**	0.0	**+36.9**	**+56.5**	+4.9	**+49.2**	-1.0
	"CTX-susceptible isolates" compared to "all isolates"	**-12.2**	**-8.2**	0.0	-4.6	**-7.0**	-0.7	**-5.9**	+0.3
