# Supplementary material for: Analysis and Presentation of Cumulative Antimicrobial Susceptibility Test Data – The Influence of Different Parameters in a Routine Clinical Microbiology Laboratory
Source: PLoS One. 2016 Jan 27;11(1):e0147965. doi: 10.1371/journal.pone.0147965 (PMC4729434; doi:10.1371/journal.pone.0147965)
Supplement: S7 Table — Cumulative antibiograms were calculated either at genus level or at species level, as detailed in the respective results and discussion section of the manuscript. In addition to the resistance rates for selected species/antibiotic combinations and the total number (n) of isolates included, the difference in resistance estimates between the different calculation approaches is shown (highlighted in light grey, with differences ≥5 percentage points in bold). (PDF) [file pone.0147965.s007.pdf]

S7 Table. Genus-specific compared to species-specific resistance estimates.

Cumulative antibiograms were calculated either at genus level or at species level, as detailed in the respective results and discussion section of the manuscript. In addition to the resistance rates for selected species/antibiotic combinations and the total number (n) of isolates included, the difference in resistance estimates between the different calculation approaches is shown (highlighted in light grey, with differences ≥5 percentage points in bold).

		Resistance rate and difference in resistance estimates, respectively (in %)		
		AMP	LVX	VAN
2013	All enterococci, n=3888	27.0	51.2	3.8
	Enterococcus faecalis only, n=2717	0.0	35.5	0.0
	Enterococcus faecium only, n=1075	89.6	89.9	8.9
	"*Enterococcus faecalis* only" compared to "all enterococci"	**-27.0**	**-15.7**	-3.8
	"*Enterococcus faecium* only" compared to "all enterococci"	**62.6**	**38.7**	**5.1**
2014	All enterococci, n=4159	28.1	47.9	3.0
	Enterococcus faecalis only, n=2964	0.0	30.0	0.0
	Enterococcus faecium only, n=1112	89.4	88.5	8.0
	"*Enterococcus faecalis* only" compared to "all enterococci"	**-28.1**	**-17.9**	-3.0
	"*Enterococcus faecium* only" compared to "all enterococci"	**61.3**	**40.6**	**5.0**
