# Supplementary material for: Analysis and Presentation of Cumulative Antimicrobial Susceptibility Test Data – The Influence of Different Parameters in a Routine Clinical Microbiology Laboratory
Source: PLoS One. 2016 Jan 27;11(1):e0147965. doi: 10.1371/journal.pone.0147965 (PMC4729434; doi:10.1371/journal.pone.0147965)
Supplement: S8 Table — Susceptibility rates, non-resistance rates and resistance rates of Escherichia coli, Klebsiella pneumoniae and Proteus mirabilis isolates were calculated before (2013) and after change in breakpoints for antimicrobial susceptibility testing (Apr-Dec 2014), as detailed in the respective results and discussion section of the manuscript. The change in estimates from 2013 to Apr-Dec 2014 is also shown (highlighted in light grey, with differences resulting from the change in breakpoints in bold). (PDF) [file pone.0147965.s008.pdf]

S8 Table. Effect of a change in breakpoints for antimicrobial susceptibility testing.

Susceptibility rates, non-resistance rates and resistance rates of *Escherichia coli*, *Klebsiella pneumoniae* and *Proteus mirabilis* isolates were calculated before (2013) and after change in breakpoints for antimicrobial susceptibility testing (Apr-Dec 2014), as detailed in the respective results and discussion section of the manuscript. The change in estimates from 2013 to Apr-Dec 2014 is also shown (highlighted in light grey, with differences resulting from the change in breakpoints in bold).

E. coli n=8754 (in 2013); n=8146 (in Apr-Dec 2014)	AMP			SAM			CXM			CTX		
	2013	2014	change	2013	2014	change	2013	2014	change	2013	2014	change
Susceptibility rate (in %)	43.2	0.0	**-43.2**	52.4	0.0	**-52.4**	79.3	0.0	**-79.3**	85.0	84.9	-0.1
Non-resistance rate (in %)	43.2	44.1	0.9	55.0	52.4	-2.6	79.4	79.6	0.2	85.0	84.9	-0.1
Resistance rate (in %)	56.8	55.9	-0.9	45.0	47.6	2.6	20.6	20.4	-0.2	15.0	15.1	0.1
K. pneumoniae n=1644 (in 2013); n=1513 (in Apr-Dec 2014)	AMP			SAM			CXM			CTX		
	2013	2014	change	2013	2014	change	2013	2014	change	2013	2014	change
Susceptibility rate (in %)	0.0	0.0	0.0	61.8	0.0	**-61.8**	76.3	0.0	**-76.3**	83.2	88.2	5.0
Non-resistance rate (in %)	0.0	0.0	0.0	69.1	74.1	5.0	76.6	79.7	3.1	83.2	88.2	5.0
Resistance rate (in %)	100.0	100.0	0.0	30.9	25.9	-5.0	23.4	20.3	-3.1	16.8	11.8	-5.0
P. mirabilis n=1483 (in 2013); n=1328 (in Apr-Dec 2014)	AMP			SAM			CXM			CTX		
	2013	2014	change	2013	2014	change	2013	2014	change	2013	2014	change
Susceptibility rate (in %)	67.2	0.0	**-67.2**	85.2	0.0	**-85.2**	96.8	0.0	**-96.8**	98.6	98.9	0.3
Non-resistance rate (in %)	67.2	66.9	-0.3	88.2	86.8	-1.4	96.8	97.0	0.2	98.6	98.9	0.3
Resistance rate (in %)	32.8	33.1	0.3	11.8	13.2	1.4	3.2	3.0	-0.2	1.4	1.1	-0.3
